# Photochromic diarylethene ligands featuring 2-(imidazol-2-yl)pyridine coordination site and their iron(II) complexes

**DOI:** 10.3762/bjoc.15.235

**Published:** 2019-10-15

**Authors:** Andrey G Lvov, Max Mörtel, Anton V Yadykov, Frank W Heinemann, Valerii Z Shirinian, Marat M Khusniyarov

**Affiliations:** 1 N. D. Zelinsky Institute of Organic Chemistry, Russian Academy of Sciences, 47, Leninsky prosp., 119991 Moscow, Russian Federation; 2 Department of Chemistry and Pharmacy, Friedrich-Alexander University Erlangen-Nürnberg (FAU), Egerlandstraße 1, 91058 Erlangen, Germany

**Keywords:** diarylethene, 2-(imidazol-2-yl)pyridine, iron(II) complex, photochromism

## Abstract

A new family of photochromic diarylethene-based ligands bearing a 2-(imidazol-2-yl)pyridine coordination unit has been developed. Four members of the new family have been synthesized. The photoactive ligands feature non-aromatic ethene bridges (cyclopentene, cyclopentenone, and cyclohexenone), as well as closely spaced photoactive and metal coordination sites aiming a strong impact of photocyclization on the electronic structure of the coordinated metal ion. The ligands with cyclopentenone and cyclohexenone bridges show good cycloreversion quantum yields of 0.20–0.32. The thermal stability of closed-ring isomers reveals half-lives of up to 20 days in solution at room temperature. The ligands were used to explore coordination chemistry with iron(II) targeting photoswitchable spin-crossover complexes. Unexpectedly, dinuclear and tetranuclear iron(II) complexes were obtained, which were thoroughly characterized by X-ray crystallography, magnetic measurements, and Mössbauer spectroscopy. The formation of multinuclear complexes is facilitated by two coordination sites of the diarylethene, acting as a bridging ligand. The bridging nature of the diarylethene in the complexes prevents photocyclization.

## Introduction

Transition metal complexes with photoactive ligands are of great interest for advanced photonic applications [1–7]. Reversible change of the electronic structure of diarylethene photochromes [8–10] upon photocyclization is a promising tool to control the electronic structure of coordinated metal ions and, consequently, associated properties. Thus, diarylethenes were integrated into well-known ligand systems, including 1,10-phenanthroline, 2-(azol-2-yl)pyridine, and related frameworks to yield photochromic ligands. The latter can be divided into two groups based on the position of the metal coordination site relative to the photoactive hexatriene unit of a diarylethene. Some diarylethene-based ligands with *pendant* coordination sites were synthesized, which allowed the remote control of luminescent, nonlinear optical and magnetic properties of transition metal complexes to some extent [11–16]. However, a *close* arrangement of hexatriene and coordination sites is the preferred approach for achieving a strong impact of the photochromic reaction on the electronic structure of a coordinated metal ion.

**Figure 1:**
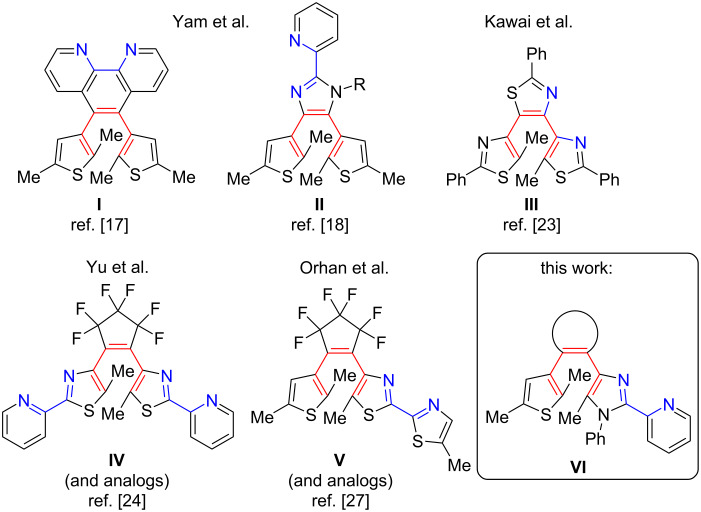
Families of diarylethene-bases ligands with spatial proximity of coordination site (blue) and photoactive framework (red).

Previously reported examples of the second group ligands are collected in Figure 1. Yam et al. developed diarylethenes **I** [17] and **II** [18] and synthesized their rhenium(I) complexes, which possess prominent luminescent and spectral properties, including photocyclization with visible and NIR light. Using the photochromic ligand **I**, a spin-crossover (SCO) Fe(II) complex was developed, which allowed a reversible paramagnetic (high-spin, *S* = 2) → diamagnetic (low-spin, *S* = 0) transition at the iron(II) ion at room temperature (rt) in solution via ligand photocyclization [19]. More recently, the remarkable photoswitching between high-spin and low-spin states at rt in the solid state and thin films was demonstrated [20–22]. Kawai et al. obtained a luminescent complex of europium(III) with terarylene **III** showing the photomodulation of emission intensity [23]. A number of diarylethene ligands with a perfluorocyclopentene bridge were designed. Yu and co-workers reported a series of 2-(thiazol-2-yl)pyridine derivatives [24–26]. Reversible photoinduced release and trapping of copper(II) ions was achieved with **IV**. Diarylethene **V** and its analogs were used as chemical sensors for a number of metal ions [27].

Despite of recent advances in this area, the scarcity of reported examples requires the search and design of novel photoswitchable ligands with advanced properties. In this context, it is important to specify some design principles for such ligands. Firstly, novel ligands should feature a non-aromatic ethene bridge to increase the life-time of a photoinduced closed-ring isomer [28]. Secondly, photoactive hexatriene and metal coordination sites should be close to each other.

To meet these requirements, a new family of ligands **VI** based on the 2-(imidazol-2-yl)pyridine unit as a heteroaryl moiety and various ethene bridges is presented in this work. Diarylethenes with the 2-(imidazol-2-yl)pyridine being a part of the ethene bridge were previously developed [18,29] and their complexes with rhodium(I) [18], platinum(II) [30], and iridium(III) [31] were reported. In contrast, in our work, the 2-(imidazol-2-yl)pyridine unit is used as a heterocyclic moiety of diarylethenes with cyclopentenone, cyclopentene, and cyclohexenone ethene bridges. The novel ligands have been tested in the coordination chemistry with iron(II) aiming photoswitchable SCO systems.

## Results and Discussion

### Synthesis and structure of photochromic ligands

**Scheme 1:**
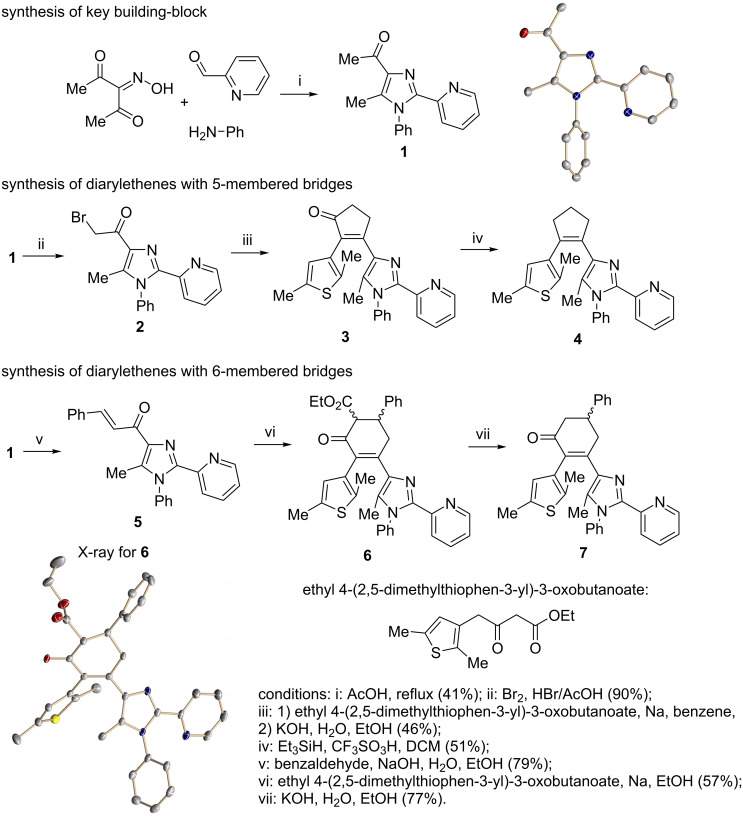
Synthesis of photochromic ligands.

Recently, some of us have developed original methods for the synthesis of various diarylethenes with cyclopentenone, cyclopentene, and cyclohexenone bridges [32]. These methods utilize ethyl 4-(2,5-dimethylthiophen-3-yl)-3-oxobutanoate (Scheme 1) or its analogs as starting materials. To obtain novel diarylethenes with 2-(imidazol-2-yl)pyridine moiety, here we have synthesized a previously unknown imidazole derivative **1** by a one-pot condensation [33] of 3-(hydroxyimino)pentane-2,4-dione, aniline, and 2-pyridinecarboxaldehyde (Scheme 1). The structure of **1** was confirmed by X-ray crystallography [34]. The ketone **1** was subsequently used for the synthesis of desired photochromic ligands with cyclopentenone (**3**), cyclopentene (**4**), and cyclohexenone (**6** and **7**) bridges via intermediate bromoketone **2** and chalkone **5**. Cyclopentenone **3** was synthesized by adapting a previously reported two-step protocol [35] starting from ethyl 4-(2,5-dimethylthiophen-3-yl)-3-oxobutanoate and bromoketone **2**. Ionic hydrogenation [36] of **3** provided diarylethene **4** with a cyclopentene bridge with 51% yield. Robinson-type reaction [37] of ethyl 4-(2,5-dimethylthiophen-3-yl)-3-oxobutanoate and chalkone **5** resulted in cyclohexenone derivative **6** (57% yield). The saponification/decarboxylation of **6** gave cyclohexenone **7**. Thus, the previously developed methodology was successfully applied for the synthesis of a new family of photochromic ligands.

The structures of the synthesized diarylethene-based ligands **3**, **4**, **6**, and **7** were confirmed by ^1^H and ^13^C NMR spectroscopy and mass spectrometry. The molecular structure of **6** was additionally confirmed by X-ray crystallography. In good accordance with previous DFT calculations [37], the molecule shows exclusively antiparallel conformation [8] of the thiophene and imidazole groups of the photoactive diarylethene moiety, with the respective α-methyl groups pointing in different directions. The thiophene and imidazole rings are rotated out of the cyclohexenone plane by 47.7° and 44.1°, respectively. The distance between the reactive carbon atoms is 3.6 Å. This value is shorter than 4.2 Å, which is favorable for photocyclization [38]. The cyclohexenone moiety adopts a distorted half-chair (sofa) conformation, with the phenyl substituted carbon atom forming an out-of-plane corner.

### Photochemical studies

**Figure 2:**
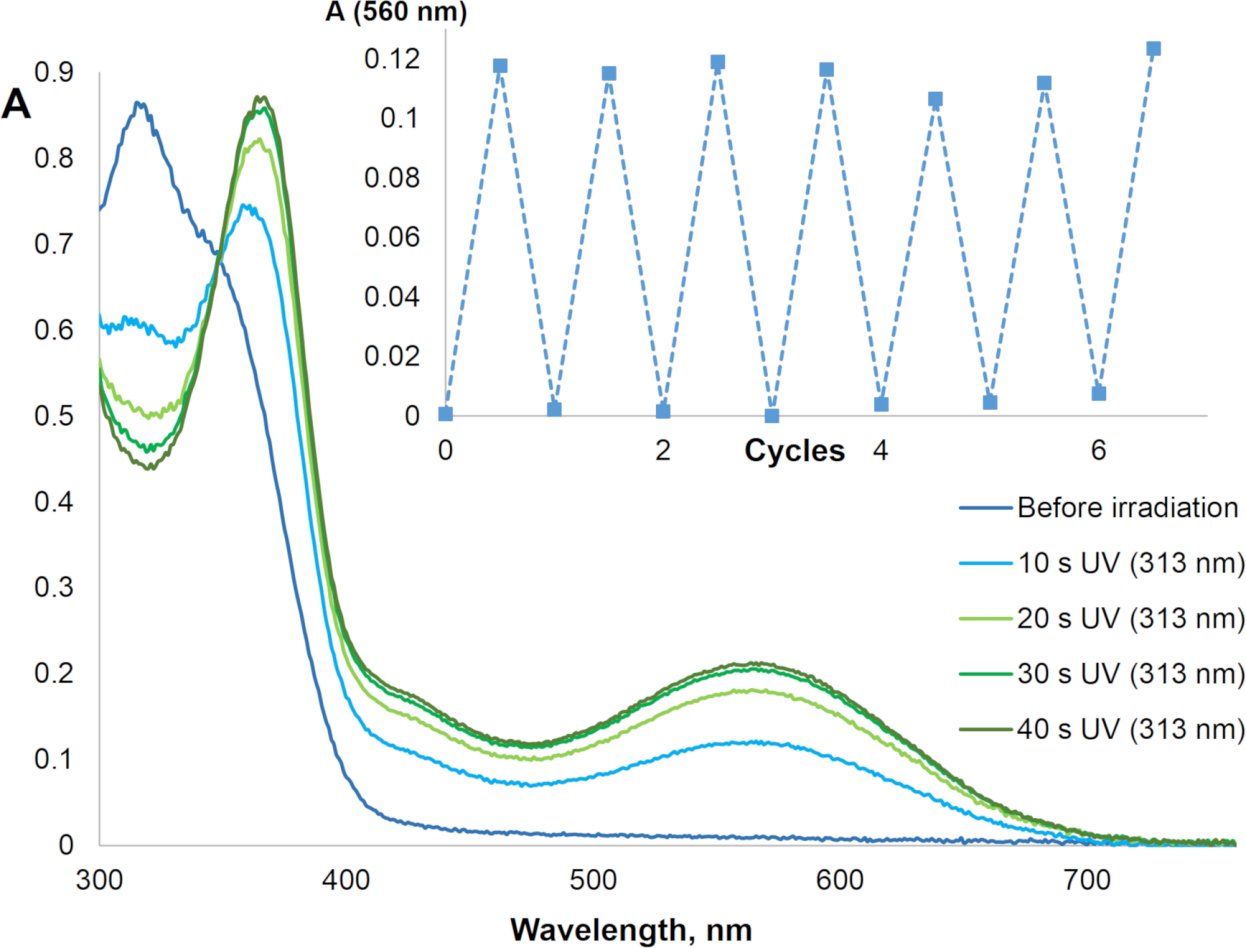
Electronic spectra of diarylethene **6** upon UV irradiation (313 nm, toluene, *c* = 3.4 × 10^−5^ M). Inset: fatigue resistance upon multiple subsequent irradiation with UV (365 nm) and visible light (green LED) in acetonitrile.

**Scheme 2:**
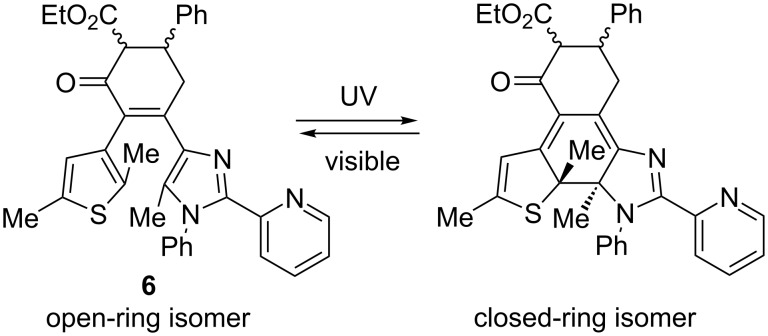
Reversible photocyclization of ligand **6**.

We have studied spectroscopic and photochemical properties of the ligands **3**, **4**, **6**, and **7** in nonpolar toluene and polar acetonitrile solvents. All ligands show typical for diarylethenes photochromic properties: colorless open-ring isomer and the emergence of a new band in visible upon UV light irradiation (Figure 2) [8]. This color change is due to the reversible photocyclization and formation of a closed-ring isomer (Scheme 2).

The results of photochemical studies are listed in Table 1. Absorption maxima of the open-ring ligands are in the 310–323 nm range. Interestingly, the carbocycle size and the presence/absence of carbonyl group are weakly reflected in the position of absorption maxima of open-ring isomers. For example, the reduction of carbonyl group in **3** (compared to **4**) leads to insignificant bathochromic shift of the absorption maximum from 322 nm to 323 nm in toluene, which is, however, accompanied by a large hypochromic shift from 31100 to 20000 M^–1^cm^–1^. The expansion from cyclopentenone to cyclohexenone bridge gives a similar effect.

**Table 1:** Spectroscopic and photochemical properties of photochromic ligands.

Diarylethene	Solvent	λ_max_^A^, nm (ε, M^−1^·cm^−1^)^a^	λ_max_^B^, nm (ε, M^−1^·cm^−1^)^b^	Φ_AB_^c^	Φ_BA_^d^	Conv at PSS^e^	τ^BA^_1/2_ (days)^f^

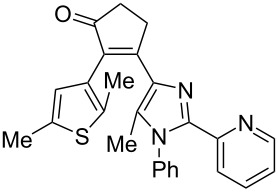 **3**	toluene	322 (31100)	572 (7000)	0.40	0.32	0.93	19.4
	MeCN	319 (31900)	563 (6700)	0.35	0.22	0.91	8.6
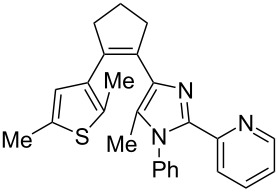 **4**	toluene	323 (20000)	504 (8400)	0.42	0.04	0.89	19.6
	MeCN	317 (19900)	490 (7300)	0.39	0.06	0.85	17.0
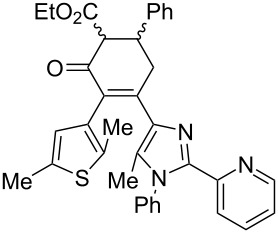 **6**	toluene	315 (22200), 346 (sh)	567 (6900)	0.25	0.29	0.89	–^g^
	MeCN	309 (18300), 341 (15500)	560 (5200)	0.26	0.20	0.91	
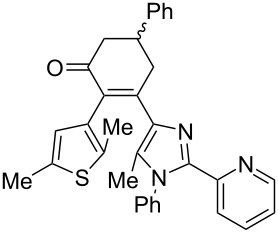 **7**	toluene	310 (24200)	560 (5600)	0.22	0.28	0.83	14.2
	MeCN	310 (18600)	549 (4800)	0.27	0.21	0.70	13.0

^a^Absorption maxima (extinction coefficients) of open-ring isomers. ^b^Absorption maxima (extinction coefficients) of closed-ring isomers. ^c^Quantum yields of photocyclization under irradiation with 313 nm. ^d^Quantum yields of cycloreversion under irradiation with 480 nm. ^e^Conversion at PSS under irradiation with 313 nm. ^f^Thermal stability of the closed-ring isomer – half-life at 25 ºC (days). ^g^Additional thermal process of photoinduced form was detected.

Cyclopentene derivative **4** shows an absorption maximum of the closed-ring isomer at 504 nm in toluene, while a hypsochromic shift of 14 nm was observed in acetonitrile. This influence of solvent polarity was detected for all ligands [39]. The carbonyl group in the ethene bridge of **3** causes a significant bathochromic shift (68 nm in toluene) of the closed-ring isomer maximum in comparison with **4**. The expansion of the five-membered carbocyclic bridge to a six-membered ring in **7** leads to a slight hypsochromic shift. The presence of a CO_2_Et group in the cyclohexenone bridge in **6** results in further minor hypsochromic shift. Thus, the absorption maxima of the closed-ring ligands are located in the wide range 490–563 nm. Photocyclization/cycloreversion of all ligands can be repeated several times without notable fatigue (see inset of Figure 2).

Extinction coefficients of closed-ring isomers were determined using ^1^H NMR and electronic absorption spectroscopy (for representative NMR spectra, see section VI in Supporting Information File 1). This allowed us to determine the quantum yields of photochemical reactions. Cyclopentene (**4**) and cyclopentenone (**3**) derivatives show the highest photocyclization quantum yields up to 0.42 in toluene. In polar acetonitrile the cyclization quantum yield is lower [40]. The quantum yields for cyclohexenone derivatives **6** and **7** are in the 0.22–0.27 range.

Similar to some other cyclopentene derivatives [41], diarylethene **4** possesses low cycloreversion quantum yields at 4–6%. However, in accordance with previous results on imidazole derivatives [42], **3** with a cyclopentenone bridge shows much high quantum yields of 0.32 and 0.22 in toluene and acetonitrile, respectively. Cyclohexenone derivatives **6** and **7** show high cycloreversion quantum yields 0.20–0.29, too. Note, that in comparison with common cyclopentene and perfluorocyclopentene derivatives [8], ligands **3**, **6**, and **7** possess high cycloreversion quantum yields. Note, that the photostationary state for the cyclization with λ = 313 nm is characterized by a high conversion to the close-ring isomers at 0.70–0.93.

Finally, thermal stability of closed-ring isomers was determined. Previously, it was demonstrated that imidazole as a heteroaryl moiety decreases thermal stability [42]. In particular, a close analogue of **3** with a phenyl instead of a pyridyl group showed a half-live of the ring-closed isomer as low as 7.3 days. In accordance to this data, diarylethene **3** showed a half-life of 8.6 days. However, in nonpolar toluene the half-life was much higher (19.4 days). Cyclopentene and cyclohexenone derivatives **4** and **7** show similar values of thermal stability (19.6 h and 14.2 h in toluene). Interestingly, we have detected an unexpected thermal process for the closed-ring isomer of **6** in the dark, resulted in the hypsochromic shift of long-wavelength absorption band, which could be due to keto–enol tautomerization in the ethene bridge [43]. Overall, the thermal stabilities of the new ligands are comparable to those of phenanthroline-based photochromic ligand **I** [19].

### Synthesis and structure of iron complexes

Previously, ligand **I** (Figure 1) and its analogs were successfully used in the synthesis of SCO [Fe^II^(H_2_B(pz)_2_)_2_**I**] complexes (pz = 1-pyrazolyl) [19–22,44]. We tested the ligands **3**, **4**, **6**, and **7** for the synthesis of analogous complexes aiming at efficient light-induced SCO at rt. In all cases, the mixing of in situ-prepared “Fe(H_2_B(pz)_2_)_2_“ with the ligands in MeOH resulted in the formation of red solutions. For ligand **6** bearing a CO_2_Et group, almost instant precipitation was observed. In contrast to **6**, our attempts to obtain crystalline solids using **3**, **4**, and **7** were unsuccessful.

**Figure 3:**
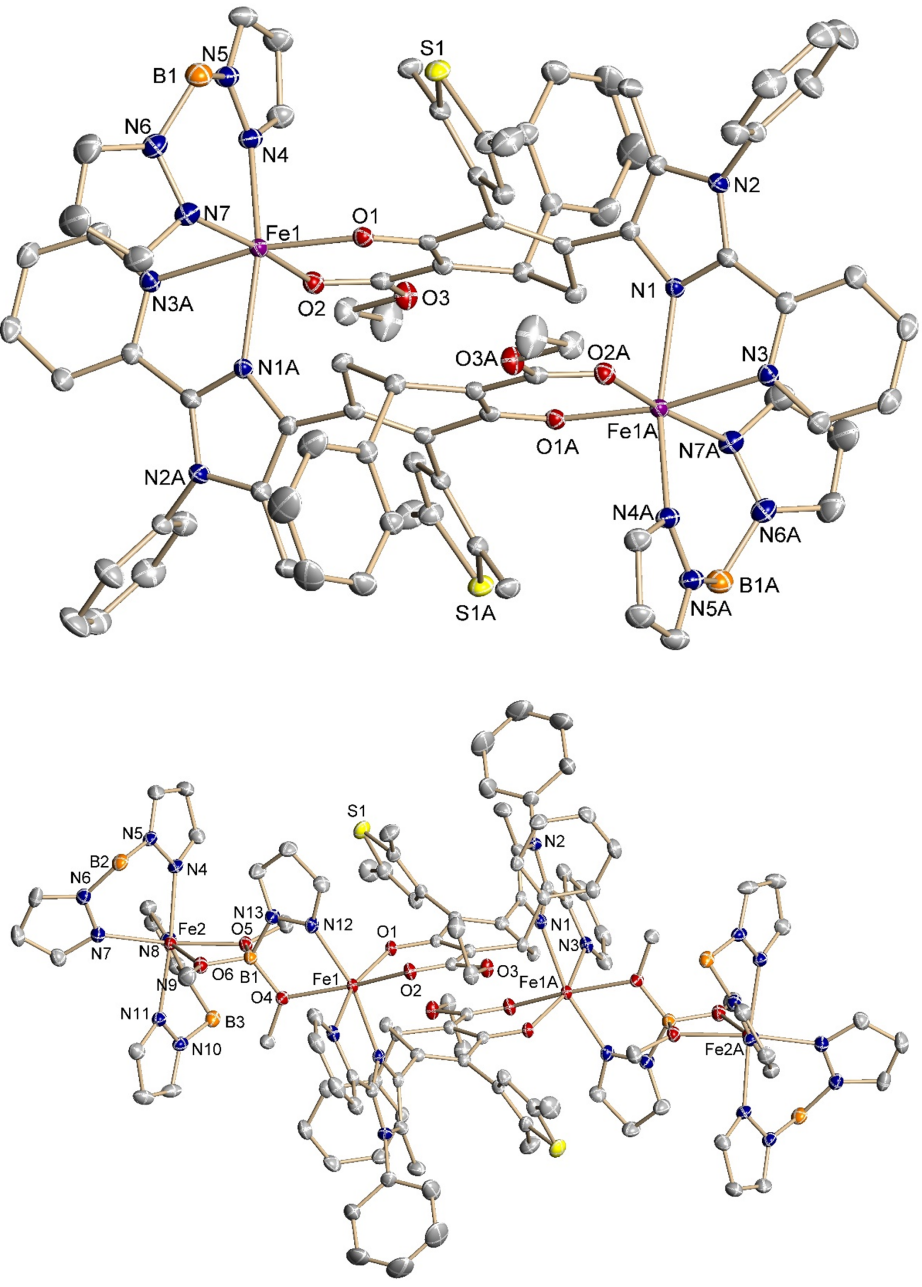
Molecular structure of complexes **8** (top) and **9** (bottom) at 100 K. The H atoms are omitted for clarity; the thermal ellipsoids are drawn at the 50% probability level.

Crystals suitable for X-ray structure were obtained in a fritted U-shape tube by the slow diffusion of methanol solutions of in situ-prepared “Fe(H_2_B(pz)_2_)_2_“ and diarylethene **6**. Unexpectedly, we have found that the structure of the product depends on the reaction time. Reproducible crystals isolated after growing for 1 month and less – a dinuclear complex **8**, and unique crystals after 2-year storage – a tetranuclear complex **9**, have different molecular structures (Figure 3). Both complexes represent unusual cyclic complexes, comprising two diarylethene ligands and two or four iron(II) ions in **8** and **9**, respectively. The β-keto ester fragment in the bridge of **6** serves as a second coordination site, which eliminates a proton under the action of H_2_B(pz)_2_^1−^ as base in both complexes [45].

The complex **8** ([Fe_2_(H_2_B(pz)_2_)_2_(**6**-H)_2_]·4CH_3_OH) crystallizes as red blocks in the monoclinic *C*2/*c* space group with four molecules of **8** and 16 molecules of co-crystallized CH_3_OH in the unit cell. The compound reveals a crystallographically symmetrical dimeric structure with two iron centers linked by two ligands **6**. Each iron ion is coordinated equatorially by the β-keto ester moiety of one molecule **6** and chelated via the imidazole and pyridine groups of another moelcule **6**. The distorted octahedral coordination sphere is completed by a H_2_B(pz)_2_^1−^ anion. Fe–N bond distances of 2.1602(15)–2.1615(15) Å for the pyrazoles and 2.2489(14)–2.2462(14) Å for the imidazole and pyridine moieties are typical for high-spin (HS) iron(II) complexes [46–47]. Similarly, Fe–O bond distances of 1.9912(11) and 2.1238(12) Å also provide evidence for HS iron(II) [46,48–49]. The two Fe–O bonds are not equivalent as the ligand appears in its deprotonated enolate form. Furthermore, a *trans*-effect due to the H_2_B(pz)_2_^1−^ anion can further elongate that bond. Two iron(II) centers are 8.057 Å apart in the dimer. The ligand **6** appears in the open-ring form and the parallel conformation [8], with the α-methyl groups pointing in similar directions. While still appearing in a distorted half-chair conformation, the phenyl substituent of the cyclohexenone moiety no longer forms the out-of-plane corner. Instead, the unsubstituted CH_2_ position twists out of the plane towards the thiophene moiety of another ligand **6**.

Long time precipitation of crystalline material resulted in a tetranuclear species [Fe_2_(H_2_B(pz)_2_)_2_Fe_2_(B(OMe)_3_(pz))_2_(**6**-H)_2_] (complex **9**). Apparently, this product is the result of destruction of bis(pyrazolyl)borate moieties of complex **8** by methanol. Species **9** crystallizes as red, block-shaped crystals in the triclinic *P−*1 space group with one molecule in the unit cell. The crystal structure reveals two pairs of differently substituted iron ions connected by a so far unprecedented trimethoxypyrazolylborate bridging group. The periphery Fe ions reveal an octahedral coordination environment with two H_2_B(pz)_2_^1−^ anions and two methoxy groups of the bridging moiety. The Fe–N bond distances ranging from 2.1361(19) to 2.1631(19) Å in **9** appear similar to those in **8**, which confirms a HS-Fe(II) ion. The Fe–O bond lengths vary between 2.1333(15) Å and 2.3110(16) Å. Curiously, the O–B bond distances to the tetrahedral boron ion do not differ in length with 1.453(3) and 1.449(3) Å, respectively.

Each of the two interior Fe ions is linked to the bridging unit via its remaining methoxy group and the pyrazole moiety. The octahedral coordination environment is completed with the β-keto ester moiety of one ligand **6** and by the imidazole and pyridine groups of the second ligand **6**. The Fe–O and Fe–N bond lengths to the bridging unit are 2.1687(15) Å and 2.1190(19) Å. The pyridine and imidazole Fe–N bond distances are 2.2467(19) Å and 2.1793(18) Å, which are very similar to those found for the dimer **8**. On the contrary, the Fe–O bond distances to the β-keto ester moiety differ much less with 2.0812(15) Å and 2.0098(15) Å. The iron–donor bond distances are in good agreement with an iron(II) HS state. The two iron(II) ions linked by the trimethoxypyrazolylborate group are 5.945 Å apart. The distance between the iron(II) ions connected by ligand **6** in **9** is elongated by 0.244 Å to 8.301 Å compared to **8**. The ligand **6** appears also in its open-ring form and parallel conformation.

### Electronic structure of complexes **8** and **9**

**Figure 4:**
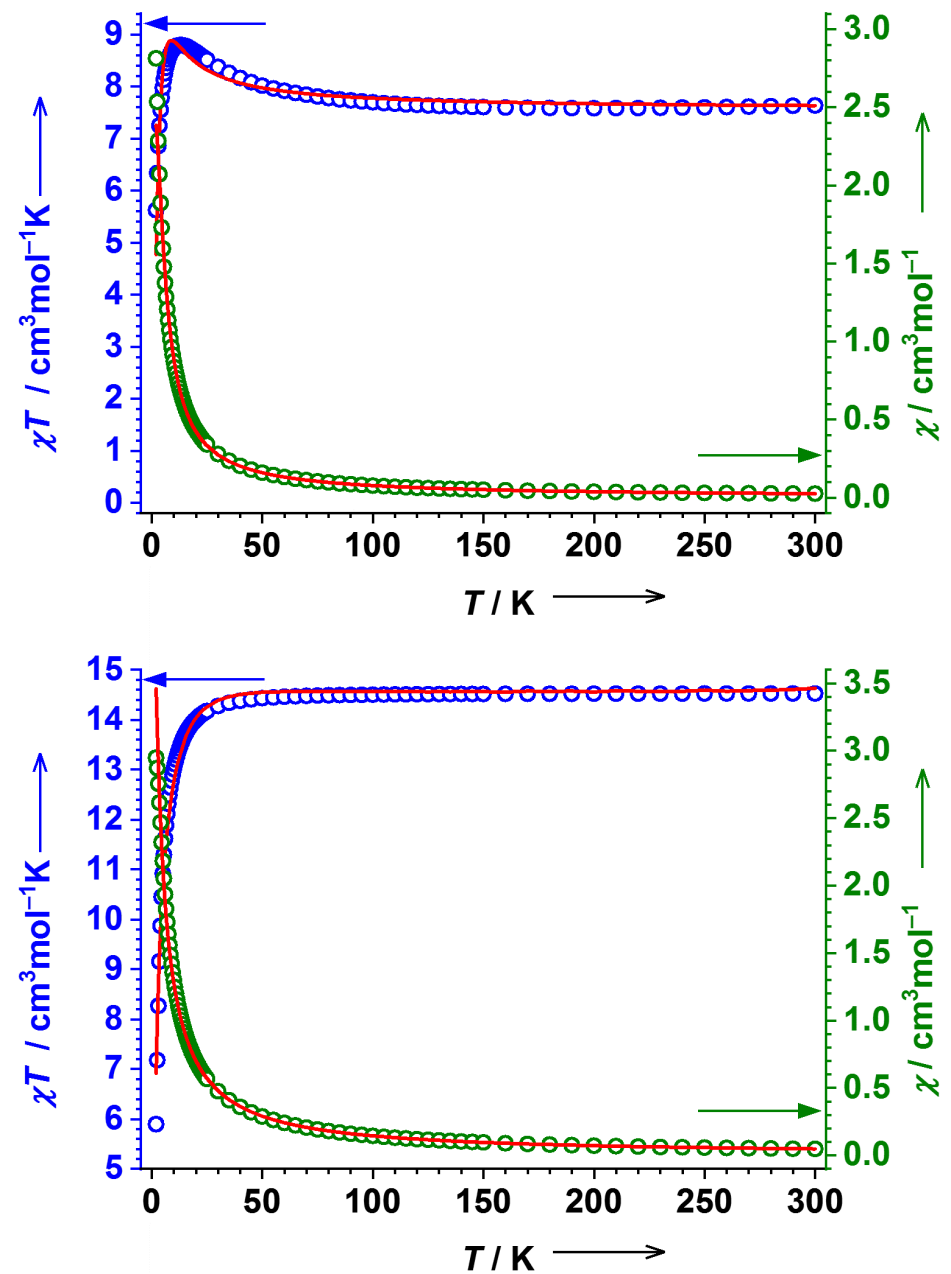
Variable temperature *χT* product (blue) and *χ* (green) of **8** (top) and **9** (bottom) measured at an external magnetic field of 1 T in the heating mode (see Supporting Information File 1 for fitting parameters).

Microcrystalline samples of **8** and **9** were used for magnetic susceptibility measurements. Complex **8** shows a nearly constant *χT* product of 7.63 cm^3^·mol^–1·^K above 125 K (Figure 4). It increases gradually upon lowering the temperature, reaching a maximum of 8.80 cm^3^·mol^–1·^K at 13 K, which is indicative of ferromagnetic coupling between the two iron(II) ions. By lowering the temperature further, the *χT* product decreases sharply due to zero-field splitting (ZFS). The rt value of the *χT* product of 7.63 cm^3^·mol^–1^·K is slightly higher than expected spin-only value for two non-interacting HS-Fe(II) ions (*χ_so_T* = 6.00 cm^3^·mol^–1·^K) due to orbital contribution. The fit for *S*_1_ = *S*_2_ = 2 spin system (see section VII in Supporting Information File 1) affords a ferromagnetic coupling constant *J* = +0.5 cm^−1^, axial ZFS *D* = 5.0 cm^−1^, and *g*_1_ = *g*_2_ = 2.24. To get further insight, field dependent measurements up to 7 T were conducted. At fields >4 T the (reduced) magnetization saturates, reaching a plateau at 8 *N*_A_µ_B_, which corresponds to two ferromagnetically coupled HS-Fe(II) ions in the ground state. Zero-field ^57^Fe Mössbauer spectrometry confirm HS-Fe(II) state at 77 K and 297 K.

The *χT* product of **9** increases to almost constant value of 14.61 cm^3^·mol^−1^·K at rt, which is in good agreement with four uncoupled HS-Fe(II) ions (*χ_so_T* = 12.00 cm^3^·mol^–1^·K) (Figure 1). A significant drop of *χT* observed at temperatures below 40 K is due to ZFS. The fit for *S*_1_ = *S*_2_ = *S*_3_ = *S*_4_ = 2 spin system yields *g* = 2.20 and *D* = 5.9 cm^−1^. Field-dependent magnetic measurements show a more gradual increase of magnetization compared to **8**. Although the magnetization does not saturate even at 7 T, the maximum of 14.17 *N*_A_µ_B_ points to four *S*_Fe_ = 2 ions (16 *N*_A_µ_B_ is expected).

The complex **8** shows a single major absorption band in the low energy UV region at 346 nm (*ε* = 6.22 × 10^4^ M^−1^·cm^−1^), which appears bathochromically shifted compared to the free ligand. No charge transfer (CT) bands are visible in the spectrum. The low intensity broad shoulder, which spans almost the entire visible region up to 750 nm, can be attributed to *d*–*d* transitions (for details, see Supporting Information File 1). The electronic absorption spectrum of **9** shows no CT bands in the visible region. The absorption maximum at 346 nm (ε = 2.28 × 10^4^ M^−1^·cm^−1^) appears sharper compared to **8**. The broad shoulder presumably due to *d*–*d* transitions also appears contracted, as it disappears at lower wavelengths (<650 nm).

Attempts to induce photocyclization of diarylethenes in complexes **8** and **9** with UV light (λ = 365 nm) in dichloromethane show no appearance of new bands. Prolonged irradiation results in slow photodecomposition. Both **8** and **9** share a common structural motif that prevents photocyclization. Coordination of the β-keto ester moiety and the imidazole/pyrazole moiety to two different iron(II) ions puts the ligand under severe strain. Thus, the structural reorganization necessary to accommodate a new planar structure formed by the photocyclization reaction is impossible without breaking Fe–N or Fe–O bonds.

Note, that the obtained complexes **8** and **9** represent cyclic complexes of diarylethenes with Fe(II) ions. In recent years, related derivatives of photochromic diarylethenes [50–51] became of great interest for realization of light-triggered guest uptake/release [52] and light-controlled interconversion between distinct supramolecular assemblies [53]. Ligand **6** featuring two coordination sites in the heteroaryl moiety and bridge provides unique opportunities to construct novel macrocyclic systems. Our future efforts will be concentrated on the synthesis of photoactive complexes based on ligand **6**.

## Conclusion

A new class of photochromic diarylethene-based ligands featuring a 2-(imidazol-2-yl)pyridine coordination unit as a heteroaryl moiety has been developed. Four members of the new family have been synthesized. All ligands show good combinations of cyclization/cycloreversion quantum yields, whereas the thermal stability of closed-ring isomers is comparable with those reported for a diarylethene-based ligand with a phenanthroline bridge. Cyclohexenone ligand **6** readily forms a dimeric complex **8** with “Fe^II^(H_2_B(pz)_2_)_2_”, with its β-keto ester moiety acting as a second coordination site. Slow crystallization yielded a tetranuclear Fe(II) complex **9**, where the base dimeric unit is expanded by an unprecedented trimethoxypyrazolylborate bridging group. The photocyclization of iron complexes is inhibited due to the rigid coordination of the imidazole group to the metal ion, which prevents the rotation of the group needed for cyclization.

## Supporting Information

File 1 Experimental details and peripheral discussion.

File 2 X-ray data for compounds **1**, **6**, **8**, and **9**.
