# Supplementary material for: Photochromic diarylethene ligands featuring 2-(imidazol-2-yl)pyridine coordination site and their iron(II) complexes
Source: Beilstein J Org Chem. 2019 Oct 15;15:2428–37. doi: 10.3762/bjoc.15.235 (PMC6808200; doi:10.3762/bjoc.15.235)
Supplement: File 1 — Experimental details and peripheral discussion. [file Beilstein_J_Org_Chem-15-2428-s001.pdf]

BEILSTEIN JOURNAL OF ORGANIC CHEMISTRY

Supporting Information

for

Photochromic diarylethene ligands featuring 2-(imidazol-2-yl)pyridine coordination site and their iron(II) complexes

Andrey G. Lvov, Max Mörtel, Anton V. Yadykov, Frank W. Heinemann, Valerii Z. Shirinian and Marat M. Khusniyarov

Beilstein J. Org. Chem. **2019,** *15,* 2428–2437. doi:10.3762/bjoc.15.235

Experimental details and peripheral discussion

License and Terms: This is a supporting information file under the terms of the Creative Commons Attribution License (http://creativecommons.org/licenses/by/4.0). Please note that the reuse, redistribution and reproduction in particular requires that the authors and source are credited.
The license is subject to the *Beilstein Journal of Organic Chemistry* terms and conditions: (https://www.beilstein-journals.org/bjoc)

Table of contents

I. General information S2
II. Synthesis of photochromic diarylethenes S4
III. Synthesis of iron(II) complexes S9
IV. Crystallographic data S10
V. Electronic spectra of photochromic diarylethenes S13
VI. ^{1}H NMR spectra during photochromic performance S17
VII. Studies of magnetic and spectral properties of complexes 8 and 9 .... S22
VIII. Copies of ^{1}H and ^{13}C NMR spectra S27
IX. References S33

I. General information

Synthesis and characterization of organic compounds. NMR spectra were recorded in deuterated solvents on Bruker AM-300 spectrometer working at 300.13 MHz for ^{1}H and 75.77 MHz for ^{13}C, respectively. Both ^{1}H and ^{13}C NMR chemical shifts are referenced relative to the residual solvents signals ($CHCl_3$: δ 7.27 for ^{1}H NMR and δ 77.16 for ^{13}C NMR) and reported in parts per million (ppm) at 293 K. Data are represented as follows: chemical shift, multiplicity (s, singlet; d, doublet; m, multiplet; br, broad), coupling constant in hertz (Hz), integration. Melting points (Mp) were recorded on a Boetius hot stage and were not corrected. High-resolution mass spectra were obtained from a TOF mass spectrometer (Bruker micrOTOF) with an ESI source.

All chemicals and anhydrous solvents were purchased from commercial sources and used without further purification. Silica column chromatography was performed using silica gel 60 (70−230 mesh); TLC analysis was conducted on silica gel 60 F_{254} plates.

Synthesis and characterization of iron(II) complexes. Elemental analyses were carried out with an EURO EA analyzer from a EuroVector. All starting materials were utilized as received without further purification unless otherwise noted. Pure anhydrous solvents were collected from a solid-state solvent purification system Glass Contour (Irvine, CA). Synthesis was performed under anaerobic conditions using Schlenk techniques.

Photochemistry of diarylethenes. UV−vis spectra were recorded with a Fluorat-Panorama spectrophotometer in 1.0 cm quartz cuvettes. The experimental measurements were performed in the presence of air in solutions of acetonitrile and toluene. The quantum yields of the photocyclization and cycloreversion (Φ_{AB} and Φ_{BA}) were measured by previously reported method [S1]. 1,2-Bis(2-methyl-1-benzothiophen-3-yl)perfluorocyclopentene in hexane solution was used as a chemical actinometer at 313 and 480 nm [S1]. The photon numbers per second at 480 nm are $\approx 7 \times 10^{15}$ photons/s; at 313 nm are $\approx 2 \times 10^{15}$ photons/s.

Molar extinction coefficients of the photogenerated isomers were determined as follows: a diarylethene (1 mg) was dissolved in appropriate deuterated solvent (0.5 ml) and the solution was irradiated with UV-light (365 nm) for 20 minutes in a NMR tube. The obtained deeply colored solution was studied by ^{1}H NMR and UV–vis spectroscopy studies (in 0.1 cm quartz cuvettes). Molar extinction coefficients (ε_c) were calculated by equation (1):

$$\varepsilon_c = \frac{D}{conv \cdot c_0} \qquad (1)$$

where D is the absorption at band maximum of photogenerated isomer; *conv* is the conversion of diarylethene; c_0 is the total concentration of the compound. For the representative ^{1}H NMR spectra with characteristic signals see Section IV.

Photochemistry of complexes. Electronic absorption spectra were recorded with a Shimadzu UV 3600 spectrophotometer. All solutions for photoexperiments were prepared under inert conditions and sealed in custom made Quartz SUPRASIL cells (QS). Irradiation experiments were conducted with the sample positioned between two opposing UV analytical lamps (Herolab GmBh, 15 W, 365 nm).

X-ray crystallographic data collection and refinement of the structures. Single crystals suitable for X-Ray diffraction were embedded in protective perfluoropolyalkyl ether oil and transferred into the cold nitrogen gas stream of the diffractometer. All sets of intensity data were collected at 120 K (compound **1**) and 100 K (compounds **6**, **8**, **9**). **1** was measured on a Bruker APEX2 CCD diffractometer (λ(Mo K_α) = 0.71072 Å). **6** was measured on a Bruker Kappa APEX 2 *IμS* Duo diffractometer equipped with QUAZAR focusing Montel optics (Mo K_α radiation; λ = 0.71073 Å). **8** was measured on a Bruker Smart APEX 2 diffractometer with a TRIUMPH curved graphite monochromator (Mo K_α radiation; λ = 0.71073 Å). **9** was measured on a Bruker Kappa Photon II *IμS* Duo diffractometer equipped with QUAZAR focusing Montel optics (Mo K_α radiation; λ = 0.71073 Å). Additional multiple measurements of equivalent reflexes for a semi-empiric absorption correction have been executed using SADABS [S2]. The structures were solved by direct methods (SHELXTL NT 6.12) [S3] and have been refined by full-matrix least squares procedures on F^2 using SHELXL 2016/6 [S4]. Hydrogen atoms were placed in a position of optimized geometry and their isotropic displacement parameters were tied to those of their corresponding carrier atoms by a factor of 1.2 or 1.5. The crystallographic data, data collection and structure refinement details are summarized in Table S1.

Magnetic studies. Magnetic susceptibility data on solid samples were collected with a Quantum Design MPMS 3 Magnetometer. DC susceptibility data were collected in the temperature range 2–300 K on powder samples restrained within a polycarbonate gel capsule in the applied magnetic field of 1 T at 1 K min^{-1} heating/cooling rate and 0.5 K intervals between 2–25 K, 5 K intervals between 25–150 K and 10 K intervals between 150–300 K. The magnetic susceptibility data were corrected for diamagnetism using an estimation $\chi_{\text{m, diamag}} = ½\ M_\text{w}\cdot 10^{-6}$ cm^{3}mol^{-1} with M_w being the molar mass of the compound [S5]. The program PHI was used for the quantitative analysis of magnetic data [S6].

^{57}Fe Mossbauer spectrometry. ^{57}Fe Mossbauer spectra were recorded on a WissEl Mossbauer spectrometer (MRG-500) in constant-acceleration mode. ^{57}Co/Rh was used as the radiation source. The temperature of the samples was controlled by an MBBC-HE0106 Mossbauer He/N_2 cryostat within an accuracy of ±0.3 K. Isomer shifts were determined relative to α-iron at 298 K. The program *MFIT* was used for the quantitative analysis of spectra [S7].

II. Synthesis of photochromic diarylethenes

The synthesis of desired photochromic ligands **3**, **4**, **6** and **7** are depicted at Scheme S1. Ethyl 4-(2,5-dimethylthiophen-3-yl)-3-oxobutanoate was synthesized according previously reported method [S8].

Scheme S1. Synthesis of diarylethenes **3**, **4**, **6**, **7**.

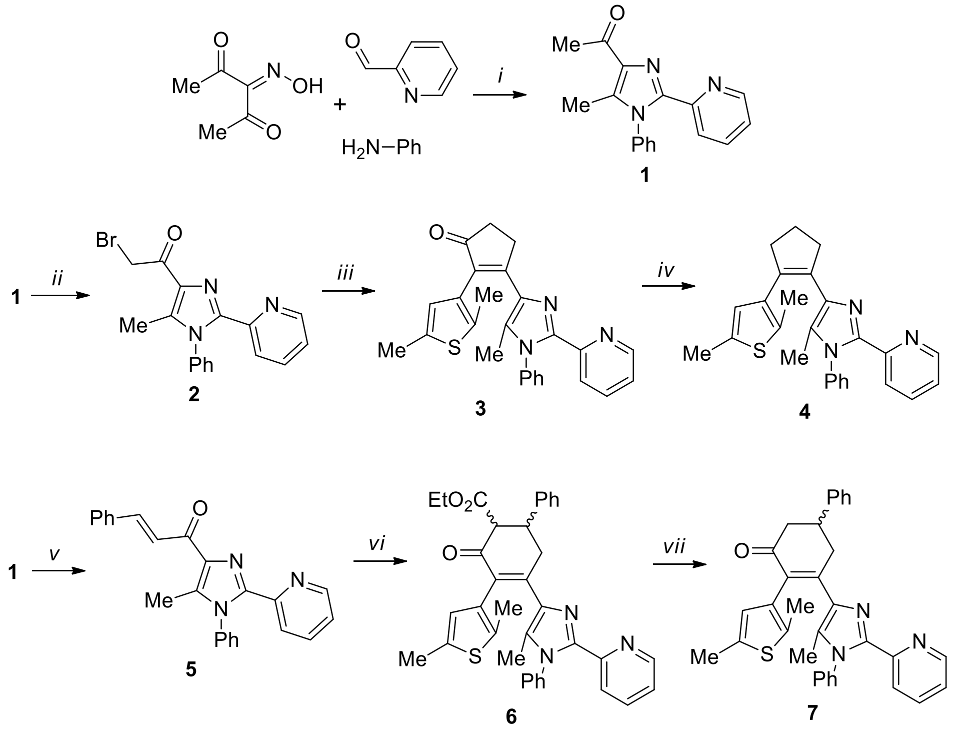

Conditions: *i*: AcOH, reflux (41%); *ii*: Br_2, HBr/AcOH (90%);
iii: 1) Ethyl 4-(2,5-dimethylthiophen-3-yl)-3-oxobutanoate, Na, benzene,
2) KOH, H_2O, EtOH (46%);
iv: Et_3SiH, CF_3SO_3H, DCM (51%);
v: benzaldehyde, NaOH, H_2O, EtOH (79%);
vi: Ethyl 4-(2,5-dimethylthiophen-3-yl)-3-oxobutanoate, Na, EtOH (57%); *vii*: KOH, H_2O, EtOH (77%).

Synthesis of imidazole 1.

3-(Hydroxyimino)pentane-2,4-dione (5.00 g, 38.7 mmol) was dissolved in acetic acid (70 mL). Aniline (4.00 mL, 42.6 mmol) and 2-pyridinecarboxaldehyde (4.20 mL, 42.6 mmol) were added to the solution and the resulting mixture was heated for reflux for 4 h. The reaction mixture was cooled to 10 °C and zinc powder (3.77 g, 58.1 mmol) was added portionwise. The resulting mixture was heated to reflux for 1 h. The resulting solution was poured into water (500 mL) and extracted with ethyl acetate (5 × 50 mL). The combined organic phases were washed with water (500 mL), dried over $MgSO_4$, and evaporated in vacuum. The residue was purified by flash chromatography with petroleum ether/ethyl acetate (2:1) to give the titled product as colorless crystals.

1-(5-Methyl-1-phenyl-2-(pyridin-2-yl)-1*H*-imidazol-4-yl)ethanone (1).

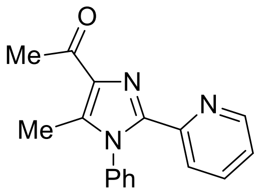

1

Colorless crystals, 41% yield (4.40 mg), mp 105-107 °C.

^{1}H NMR (300 MHz, $CDCl_3$): δ = 2.42 (s, 3H), 2.72 (s, 3H), 7.10-7.20 (m, 3H), 7.45-7.49 (m, 3H), 7.66 (td, J = 7.8, 1.5 Hz, 1H), 7.80 (d, J = 7.9 Hz, 1H), 8.32 (d, J = 4.4 Hz, 1H).

^{13}C NMR (75 MHz, $CDCl_3$): δ = 11.2, 27.7, 123.0, 123.5, 127.6 (2C), 128.9, 129.3 (2C), 136.4, 136.8, 136.9, 137.7, 144.4, 148.8 (2C), 196.4.

HRMS (ESI-TOF) *m/z* $[M+H]^+$ Calcd for $C_{17}H_{16}N_3O$ 278.1288; Found 278.1293.

Synthesis of bromoketone 2.

2-Bromo-1-(5-methyl-1-phenyl-2-(pyridin-2-yl)-1*H*-imidazol-4-yl)ethanone (2).

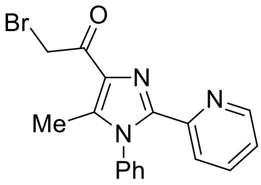

2

Imidazole **1** (1.00 g, 3.6 mmol) was dissolved in 33% solution of HBr in AcOH (5 mL) and bromine (0.19 ml, 3.6 mmol) was added. After the completion of the reaction (TLC control), the mixture was poured into water (100 mL), extracted with dichloromethane (2 × 50 mL), washed with water (100 mL), dried over $MgSO_4$, and evaporated in vacuum. The resulting product was used in the next step without further purification.

Synthesis of diarylethene 3.

This compound was synthesized in a similar manner as described in [S9].

To a solution of ethyl 4-(2,5-dimethylthiophen-3-yl)-3-oxobutanoate (1.00 g, 4.2 mmol) in dry benzene (10 mL) sodium (0.10 g, 4.4 mmol) was added, and the mixture was stirred for 3 h. To the reaction mixture a bromoketone **2** (1.50 g, 4.2 mmol) was added. The solution was stirred overnight at room temperature, then poured into cold water (150 mL) and extracted with ethylacetate (3 × 50 mL). The combined organic phases were washed with water (100 mL), and evaporated in vacuo. The residue was dissolved in ethanol (16 mL) and a solution of KOH (1.18 g, 21 mmol) in water (16 mL) was added. The reaction mixture was refluxed for 4 h, then cooled, poured into water (100 mL) and extracted with ethyl acetate (3×20 mL). The combined extracts were washed with water (50 mL), dried with magnesium sulfate and evaporated in vacuum. The residue was purified by column chromatography with petroleum ether/ethyl acetate (2:1).

2-(2,5-Dimethylthiophen-3-yl)-3-(5-methyl-1-phenyl-2-(pyridin-2-yl)-1*H*-imidazol-4-yl)cyclopent-2-enone (3).

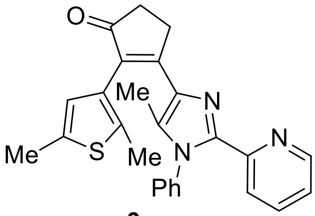

3

Pale gray powder, 46% yield (0.82 g), mp 163-165 °C.

^{1}H NMR (300 MHz, $CDCl_3$): δ = 1.45 (s, 3H), 2.06 (s, 3H), 2.38 (s, 3H), 2.63-2.72 (m, 2H), 3.25-3.33 (m, 2H), 6.58 (s, 1H), 7.06-7.16 (m, 3H), 7.37-7.45 (m, 3H), 7.60-7.67 (m, 1H), 7.79 (d, *J* = 7.9 Hz, 1H), 8.29 (d, *J* = 4.6 Hz, 1H).

^{13}C NMR (75 MHz, $CDCl_3$): δ = 10.3, 14.4, 15.2, 29.8, 34.7, 122.7, 123.2, 127.0, 127.5 (2C), 128.5, 129.2 (2C), 129.7, 132.4, 134.1, 134.7, 135.0, 135.8, 136.2, 137.5, 146.1, 148.8, 149.2, 164.8, 208.1.

HRMS (ESI-TOF) *m/z* $[M+H]^+$ Calcd for $C_{26}H_{25}N_3OS$ 426.1635; Found 426.1613.

Synthesis of diarylethene 4.

This compound was synthesized in a similar manner as described in [S10].

To a solution of diarylethene **3** (0.25 g, 0.6 mmol) in dry dichloromethane (3 mL) under inert atmosphere (argon) solutions of triethylsilane (140 mg, 1.2 mmol) in dry dichloromethane (3 mL) and trifluoromethanesulfonic acid (180 mg, 1.2 mmol) in dry dichloromethane (3 mL) were added dropwise simultaneously. The resulting emulsion refluxed until complete conversion of **3** (TLC control). The resulting solution was poured into 5% water solution of $NaHCO_3$ (100 mL) and extracted with dichloromethane (2 × 30 mL). The combined organic phases were washed with water (100 mL), dried over $MgSO_4$, and evaporated in vacuum. The residue was purified by flash chromatography by petroleum ether (100 mL, for removing of silicon-containing impurities) and petroleum ether/ethyl acetate (6:1).

2-(4-(2-(2,5-Dimethylthiophen-3-yl)cyclopent-1-en-1-yl)-5-methyl-1-phenyl-1*H*-imidazol-2-yl)pyridine (4).

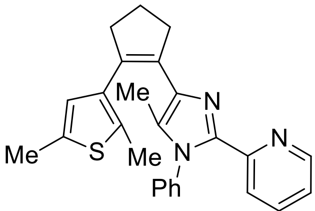

4

Pale yellow powder, 51% yield (124 mg), mp 126-129 °C.

^{1}H NMR (300 MHz, $CDCl_3$): δ = 1.44 (s, 3H), 2.04 (s, 3H), 2.05-2.14 (m, 2H), 2.37 (s, 3H), 2.81 (t, *J* = 7.3 Hz, 1H), 3.07 (t, *J* = 7.3 Hz, 1H), 6.51 (s, 1H), 7.02-7.13 (m, 3H), 7.37-7.42 (m, 3H), 7.57-7.64 (m, 1H), 7.80 (d, *J* = 7.9 Hz, 1H), 8.25 (d, *J* = 4.6 Hz, 1H).

^{13}C NMR (75 MHz, $CDCl_3$): δ = 10.0, 14.4, 15.1, 22.9, 37.5, 38.8, 122.2, 123.2, 126.4, 127.6 (2C), 128.1, 128.6, 129.0 (2C), 131.8, 132.6, 134.5, 135.1, 135.9, 136.1, 138.1, 144.4, 148.6,

HRMS (ESI-TOF) *m/z* $[M+H]^+$ Calcd for $C_{26}H_{27}N_3S$ 412.1842; Found 412.1833.

Synthesis of chalkone 5.

To a suspension of imidazole **1** (4.21 g, 15.2 mmol) and benzaldehyde (1.55 mL, 15.2 mmol) in ethanol (21 mL) solution of KOH (0.78 g, 19.5 mmol) in water (7 mL) were added. The resulting mixture was stirred at ambient temperature for 24 h and poured in water (200 mL). The precipitate was filtered off, washed with water and recrystallized from ethanol.

(*E*)-1-(5-Methyl-1-phenyl-2-(pyridin-2-yl)-1*H*-imidazol-4-yl)-3-phenylprop-2-en-1-one (5).

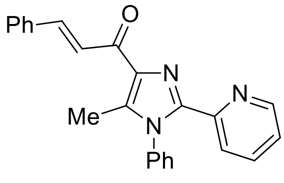

5

Yellow powder, 79% yield (4.38 mg), mp 135-137 °C.

^{1}H NMR (300 MHz, $CDCl_3$): δ = 2.51 (s, 3H), 7.14 (dd, J = 7.4, 4.9 Hz, 1H), 7.20-7.24 (m, 2H), 7.39-7.49 (m, 4H), 7.68 (td, J = 7.8, 1.6 Hz, 1H), 7.73-7.77 (m, 2H), 7.84-7.92 (m, 2H), 8.21 (d, J = 16.0 Hz, 1H), 8.31 (d, J = 4.6 Hz, 1H).

^{13}C NMR (75 MHz, $CDCl_3$): δ = 11.4, 122.9, 123.5, 123.8, 127.6 (2C), 128.7 (4C), 128.8, 129.2 (2C), 129.9, 135.6, 136.2, 137.0, 137.4, 139.3, 142.0, 144.7, 148.8, 149.1, 186.1.

HRMS (ESI-TOF) *m/z* $[M+Na]^+$ Calcd for $C_{24}H_{19}N_3ONa$ 388.1420; Found 388.1417.

Synthesis of diarylethene 6.

This compound was synthesized in a similar manner as described in [S10]. To a solution of ethyl 4-(2,5-dimethylthiophen-3-yl)-3-oxobutanoate (480 mg, 2 mmol) in dry ethanol (7 mL) metallic sodium (46 mg, 2 mmol) was added. After completion of hydrogen evolution, chalkone (600 mg, 2 mmol) was added, and the resulting suspension was refluxed. After the completion of the reaction (TLC control), the mixture was poured into water (100 mL), extracted with ethyl acetate (3 × 50 mL), washed with brine (100 mL), dried over $MgSO_4$, and evaporated in vacuum. The residue was purified by column chromatography (petroleum ester/ethyl acetate, 1:1).

Ethyl 4-(2,5-dimethylthiophen-3-yl)-5-(5-methyl-1-phenyl-2-(pyridin-2-yl)-1*H*-imidazol-4-yl)-3-oxo-1,2,3,6-tetrahydro-[1,1'-biphenyl]-2-carboxylate (6)

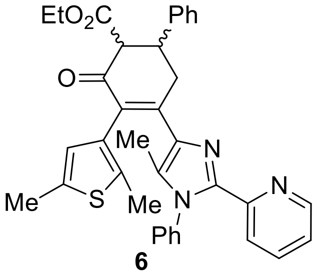

6

Pale yellow powder, 57% yield (0.67 g), mp 191-193 °C.

^{1}H NMR (300 MHz, DMSO-d_6): δ = 0.94-1.03 (m, 3H), 1.38 (s, 3H), 1.97 (s, 3H), 2.32 (s, 3H), 3.10-3.25 (m, 1H), 3.45-3.55 (m, 1H), 3.75-3.85 (m, 1H), 3.90-4.02 (m, 2H), 4.26 (d, J = 13.0 Hz, 1H), 6.32 (s, 1H), 7.00-7.14 (m, 2H), 7.19-7.51 (m, 9H), 7.72-7.90 (m, 2H), 8.16-8.20 (m, 1H).

^{13}C NMR (75 MHz, DMSO-d_6): δ = 10.6, 14.3 (2C), 15.2, 38.8, 43.4, 60.1, 60.4, 123.5, 123.8, 127.5, 127.7 (2C), 128.0 (2C), 128.7, 128.9 (2C), 129.2, 129.8 (2C), 131.6, 132.1, 134.1, 135.9, 137.3, 137.4, 142.1, 144.9, 148.4, 148.8, 152.0, 169.7.

HRMS (ESI-TOF) *m/z* $[M+H]^+$ Calcd for $C_{36}H_{34}N_3O_3S$ 588.2315; Found 588.229.

Synthesis of diarylethene 7.

This compound was synthesized in a similar manner as described in [S10].

To a solution of diarylethene **6** (0.4 mmol) in ethanol (2 mL) solution of KOH (112 mg, 2.0 mmol) in water (2 mL) was added and the resulting mixture was refluxed until completion of the reaction (TLC control). The resulting solution was poured into water (50 mL), extracted with ethyl acetate (3 × 30 mL), washed with brine (50 mL), dried over $MgSO_4$, and evaporated in vacuum. The residue was purified by column chromatography eluting by petroleum ether/ethyl acetate (2:1).

4-(2,5-Dimethylthiophen-3-yl)-5-(5-methyl-1-phenyl-2-(pyridin-2-yl)-1*H*-imidazol-4-yl)-1,6-dihydro-[1,1'-biphenyl]-3(2*H*)-one (7)

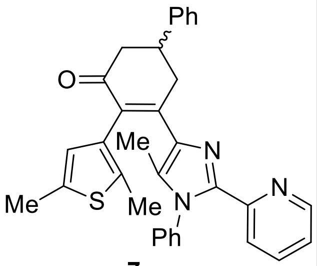

7

Yellow powder, 77% yield (158 mg), mp 171-174 °C.

^{1}H NMR (300 MHz, $CDCl_3$): δ = 1.42 (s, 3H), 2.05 (s, 3H), 2.38 (s, 3H), 2.92-3.13 (m, 3H), 3.61-3.89 (m, 2H), 6.42 (br s, 1H), 7.04-7.15 (m, 3H), 7.24-7.46 (m, 8H), 7.65 (t, J = 8.0 Hz, 1H), 7.77-7.84 (m, 1H), 8.29 (d, J = 4.7 Hz, 1H).

^{13}C NMR (75 MHz, $CDCl_3$): δ = 10.5, 14.6, 15.1, 39.6, 40.0, 45.1, 123.0, 123.6, 126.8, 126.9 (2C), 127.4 (2C), 128.2, 128.6 (2C), 129.2 (2C), 130.8, 131.7, 131.8, 134.4, 136.4, 137.2, 143.4, 148.9.

HRMS (ESI-TOF) *m/z* $[M+H]^+$ Calcd for $C_{33}H_{30}N_3OS$ 516.2104; Found 516.2083.

III. Synthesis of iron(II) complexes

These complexes were synthesized in a similar manner as described in [S11].

Synthesis of iron(II) complexes was performed under anaerobic conditions using Schlenk techniques. A solution of $K[H_2B(pz)_2]$ (127 mg, 0.7 mmol) in 3 mL of dry methanol was added to a solution of $FeSO_4 \cdot 7H_2O$ (95 mg, 0.3 mmol) in 5 mL of dry methanol and stirred at rt for 10 min. After centrifuging and filtration, a solution of diarylethene **6** (200 mg, 0.3 mmol) in 15 mL of dry methanol was added dropwise to the filtrate to give a red solution with a red powder. After 2 h, the precipitate was filtered off and washed with methanol (10 mL), toluene (3 mL) and water (20 mL) to give complex **8** as an analytically pure material.

Single crystals of complexes **8** and **9** were obtained in a fritted U-shape tube by the slow diffusion of methanol solutions of freshly prepared $Fe([H_2B(pz)_2])_2$ and diarylethene **6**. In the case of complex **8**, the U-tube was stored for 1 month. In the case of complex **9**, the U-tube was stored for 2 years.

Complex 8

Yield: 53% (268 mg).

Elem. anal. Calcd for $C_{84}H_{80}B_2Fe_2N_{14}O_6S_2$: C, 63.89; H, 5.11; N, 12.42; S, 4.06. Found: C, 63.22; H, 5.01; N, 12.13; S, 3.80.

Complex 9

Yield: 6% (41mg).

Elem. anal. Calcd for $C_{108}H_{120}B_6Fe_4N_{26}O_{12}S_2$: N, 15.65; C, 55.75; H, 5.20; S, 2.76. Found: N, 15.25; C, 56.13; H, 5.31; S, 2.77.

IV. Crystallographic Data

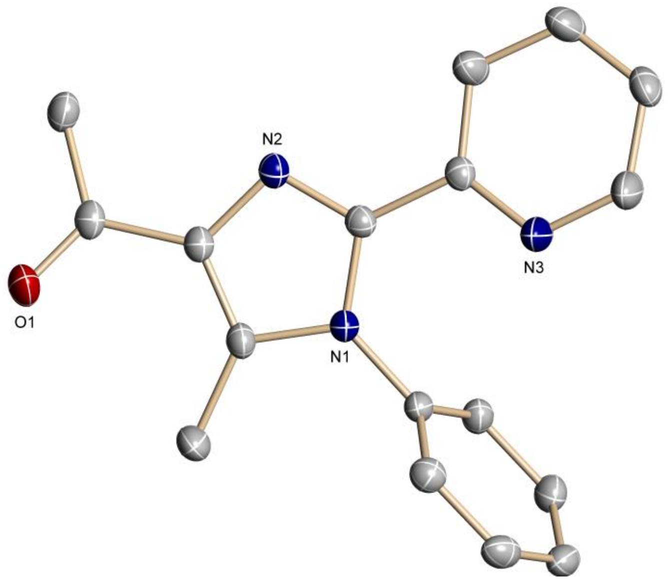

Figure S1. Molecular structure of imidazole **1** at 120 K. The H atoms are omitted for clarity; the thermal ellipsoids are drawn at the 50% probability level.

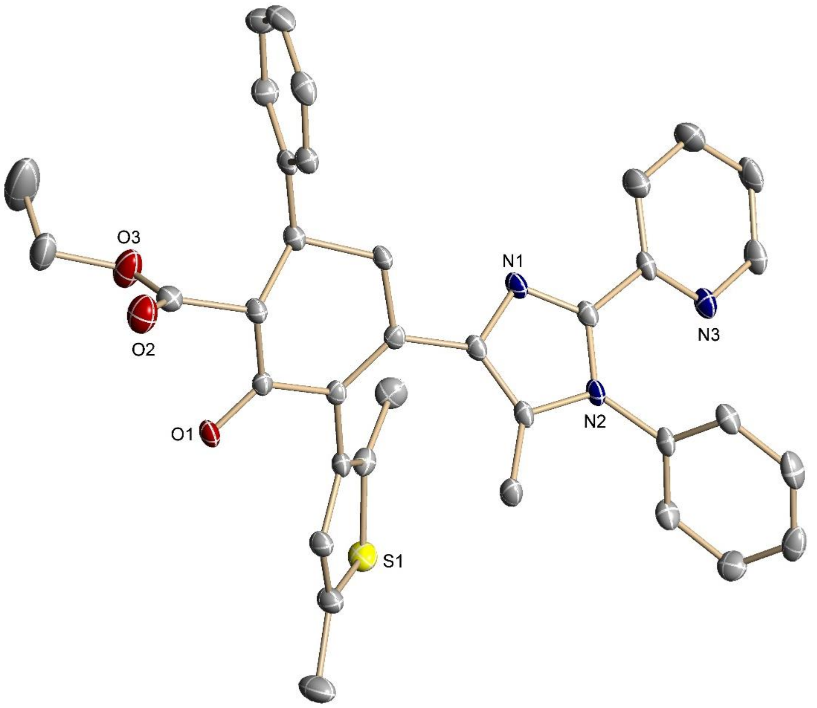

Figure S2. Molecular structure of open-ring **6** at 100 K showing an antiparallel conformation. The H atoms are omitted for clarity; the thermal ellipsoids are drawn at the 50% probability level.

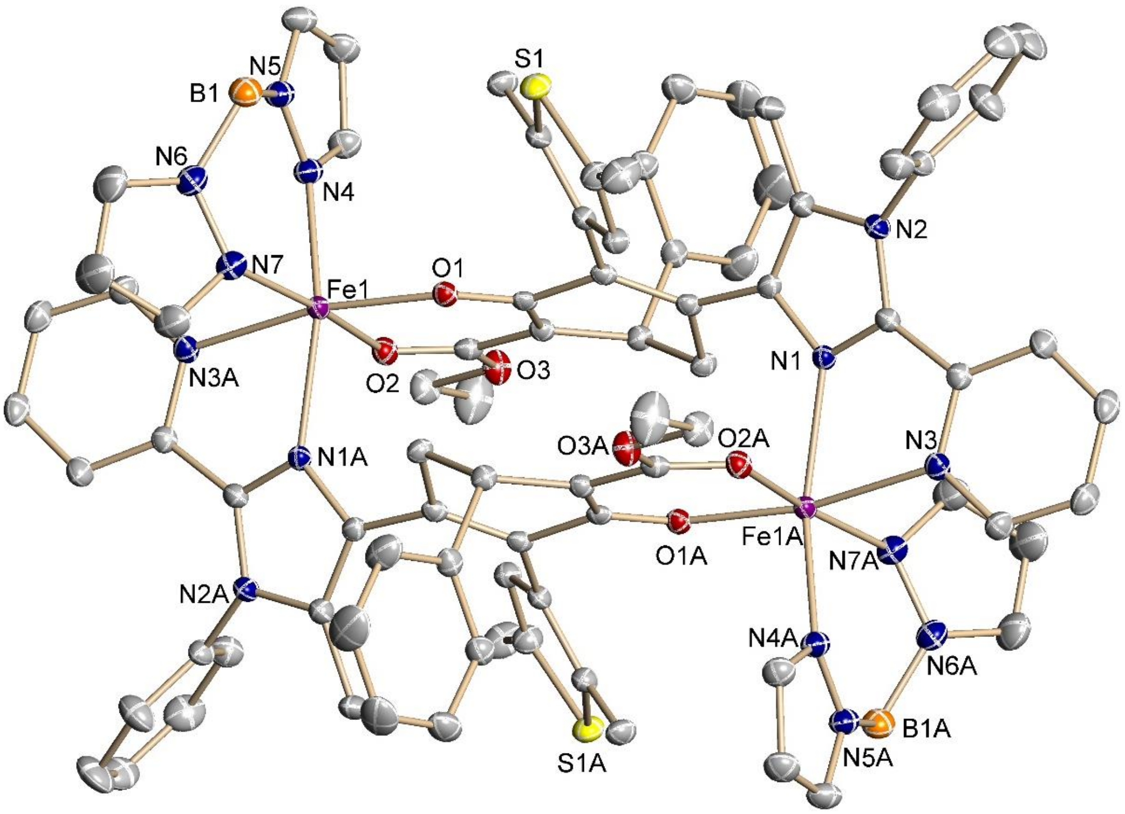

Figure S3. Molecular structure of open-ring **8** at 100 K showing a parallel conformation. The H atoms are omitted for clarity; the thermal ellipsoids are drawn at the 50% probability level.

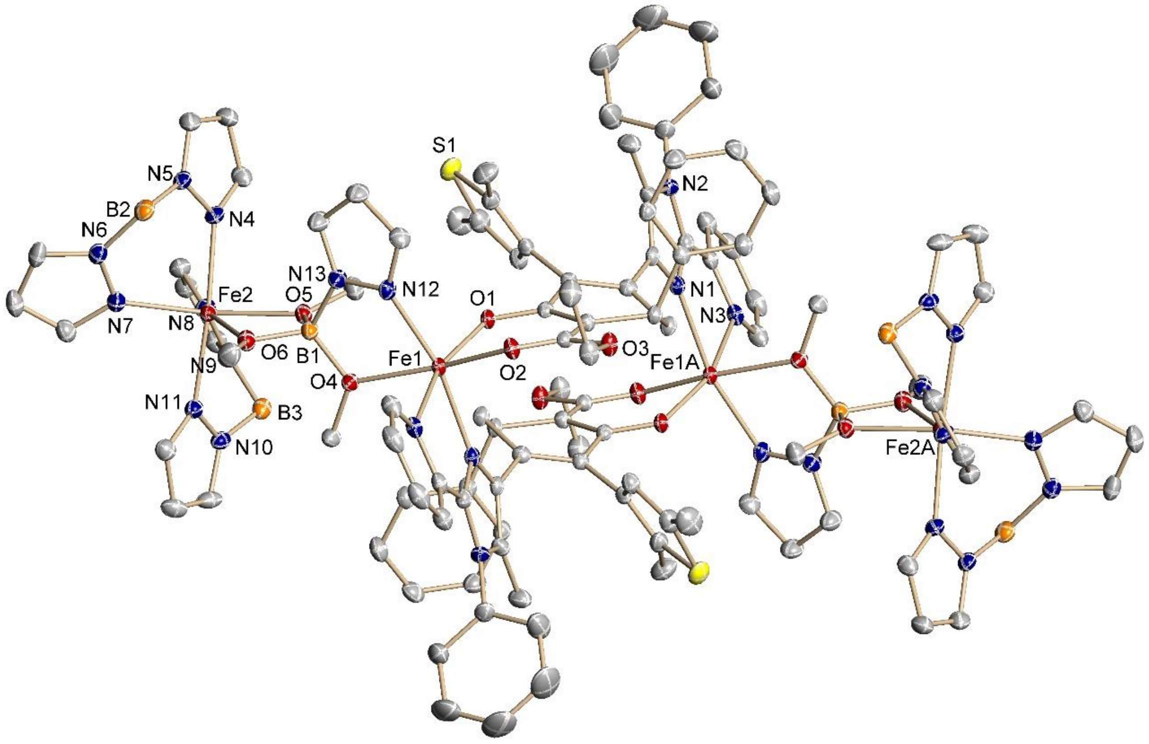

Figure S4. Molecular structure of open-ring **9** at 100 K showing a parallel conformation. The H atoms are omitted for clarity; the thermal ellipsoids are drawn at the 50% probability level.

Table S1. Crystallographic data, data collection and structure refinement details.

	**1**	**6**	**8**	**9**
Temperature, K	120(2)	100	100	100
Chemical formula	$C_{17}H_{15}N_3O$	$C_{36}H_{33}N_3S$	$C_{84}H_{80}B_2Fe_2N_{14}O_6S_2 \cdot 4(CH_3OH)$	$C_{108}H_{120}B_6Fe_4N_{26}O_{12}S_2$
Formula weight	277.32	587.22	1707.22	2326.70
Crystal size, mm	0.35×0.25×0.25	0.18×0.10×0.07	0.36×0.20×0.15	0.13×0.12×0.1
Crystal system	monoclinic	monoclinic	monoclinic	triclinic
space group	*P21/n*	$P2_1/c$	$C2/c$	$P1$
a, Å	10.7440(6)	16.936(2)	21.620(1)	12.7365(8)
b, Å	7.9933(5)	10.880(2)	17.9734(8)	15.6595(10)
c, Å	16.2596(10)	17.784(2)	23.329(2)	16.1507(10)
α, deg	90	90	90	101.570(2)
β, deg	95.0710(10)	110.925(7)	111.190(3)	109.498(2)
γ, deg	90	90	90	102.930(2)
V, Å^3	1390.91(14)	3060.8(8)	8452.4(7)	2823.6(3)
Z	4	4	4	1
ρ_{calcd}, g·cm^{-3}	1.324	1.275	1.342	1.368
reflns collected/$2\theta_{max}$, deg	27160/57.998	82688/55.8	172426/55.8	80716/55.4
unique reflns/I > 2σ(I)	27160/3336	7337/5504	10083/8440	14555/10624
no. of param/restraints	192/0	392/0	558/0	719/0
λ, Å/μ(Kα), mm^{-1}	0.71073/0.085	0.71073/0.147	0.71073/0.460	0.71073/0.61
R_1 [I > 2σ (I)]	0.0431	0.0490	0.0378	0.0473
wR_2/goodness of fit	0.1231/1.003	0.1376/1.057	0.1026/1.106	0.0934/1.033
residual density, e Å^{-3}	0.33/-0.24	+0.537/–0.446	+0.761/–0.610	+0.458/–0.598

V. Electronic spectra of photochromic diarylethenes

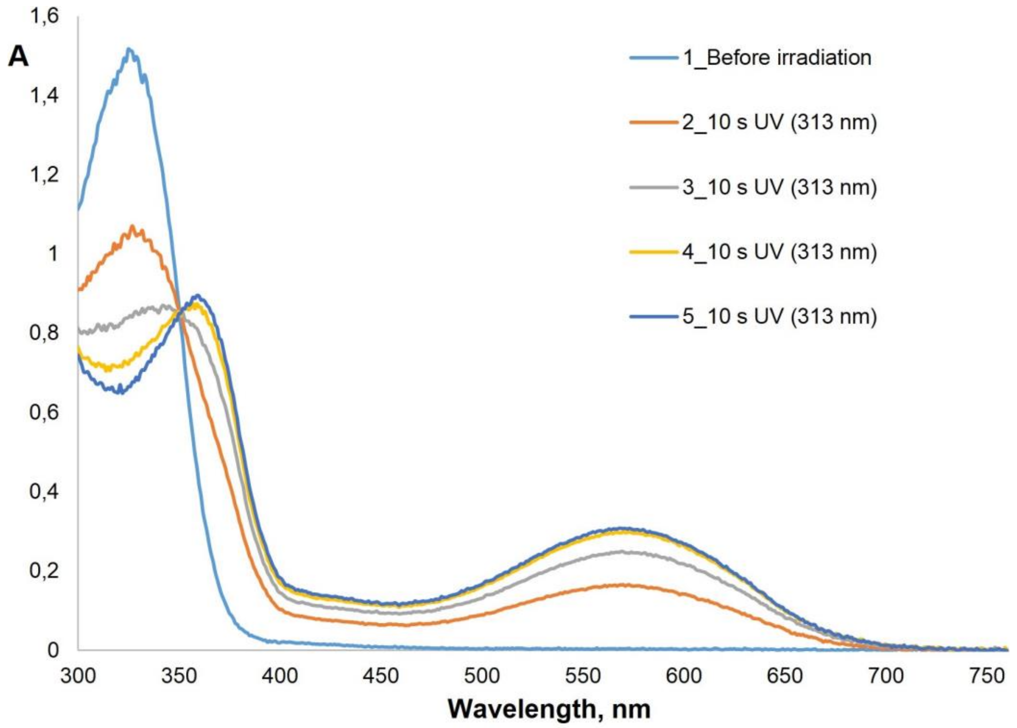

Figure S5. Electronic spectra of diarylethene **3** before and after irradiation with UV light (λ = 313 nm, toluene, $c = 4.7 \times 10^{-5}$ M).

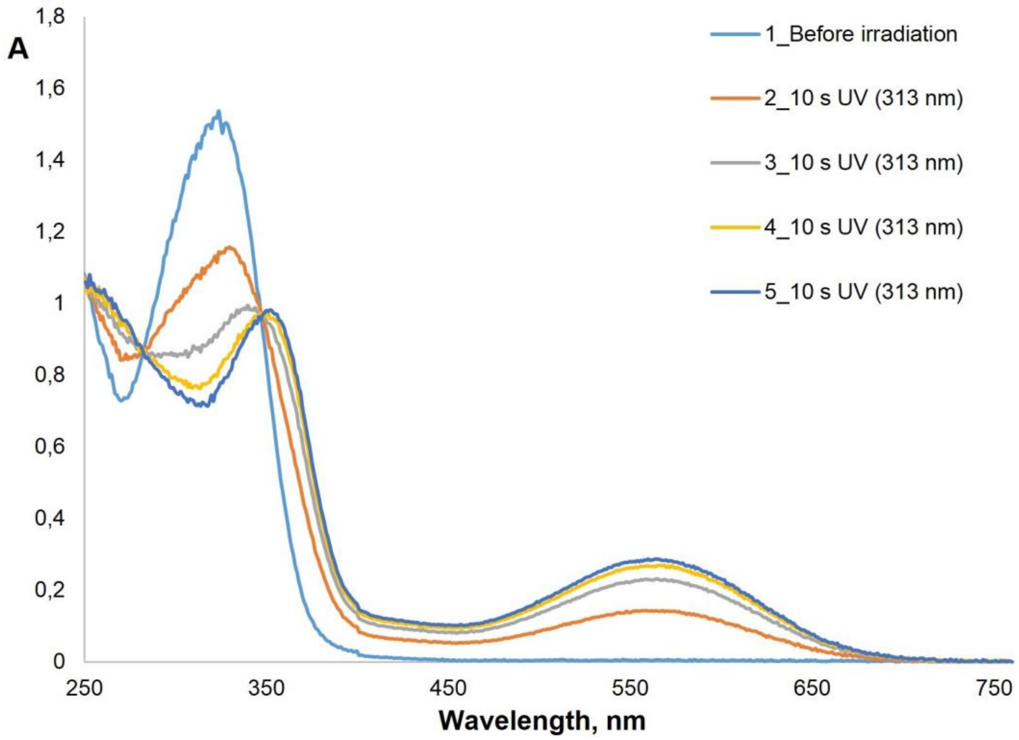

Figure S6. Electronic spectra of diarylethene **3** before and after irradiation with UV light (λ = 313 nm, acetonitrile, $c = 4.7 \times 10^{-5}$ M).

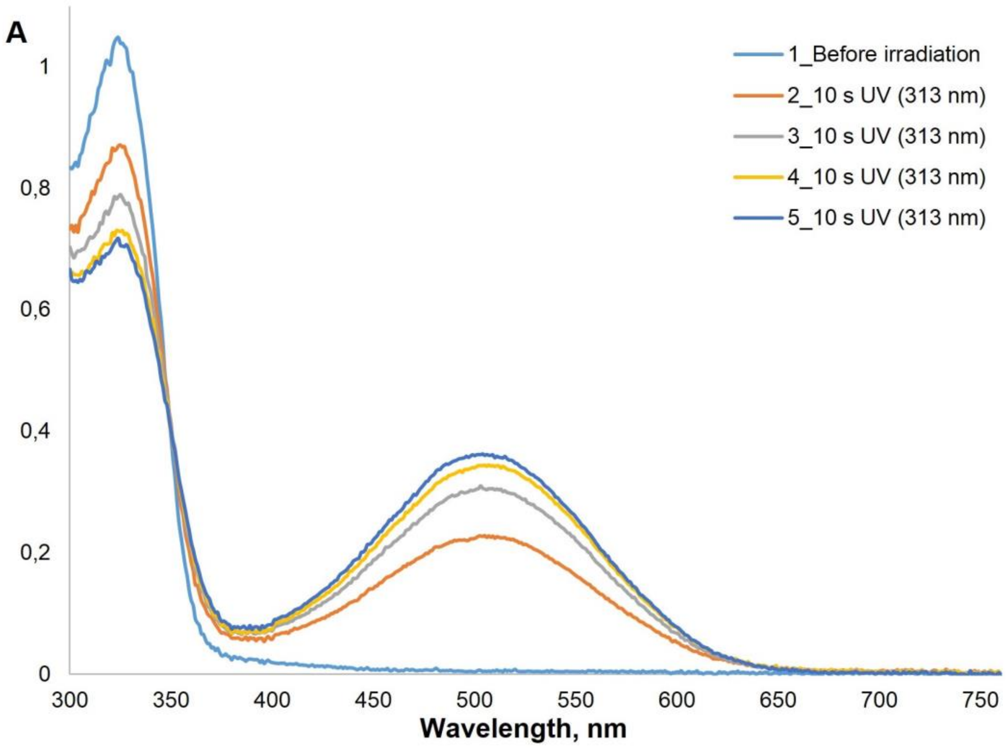

Figure S7. Electronic spectra of diarylethene **4** before and after irradiation with UV light (λ = 313 nm, toluene, $c = 4.9 \times 10^{-5}$ M).

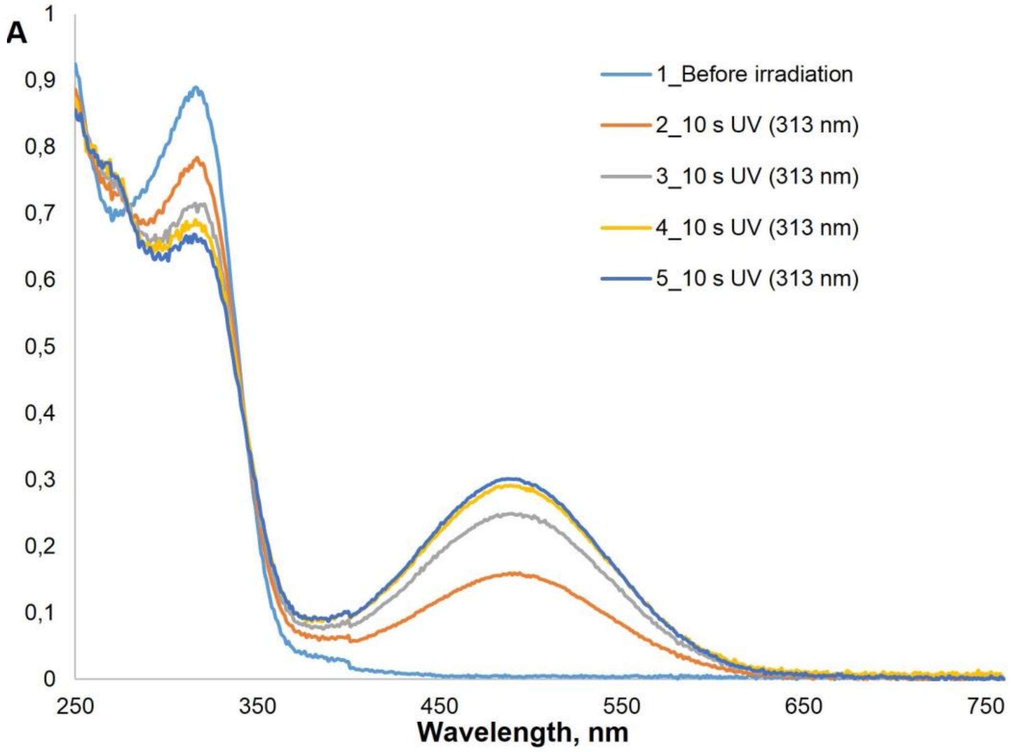

Figure S8. Electronic spectra of diarylethene **4** before and after irradiation with UV light (λ = 313 nm, acetonitrile, $c = 4.9 \times 10^{-5}$ M).

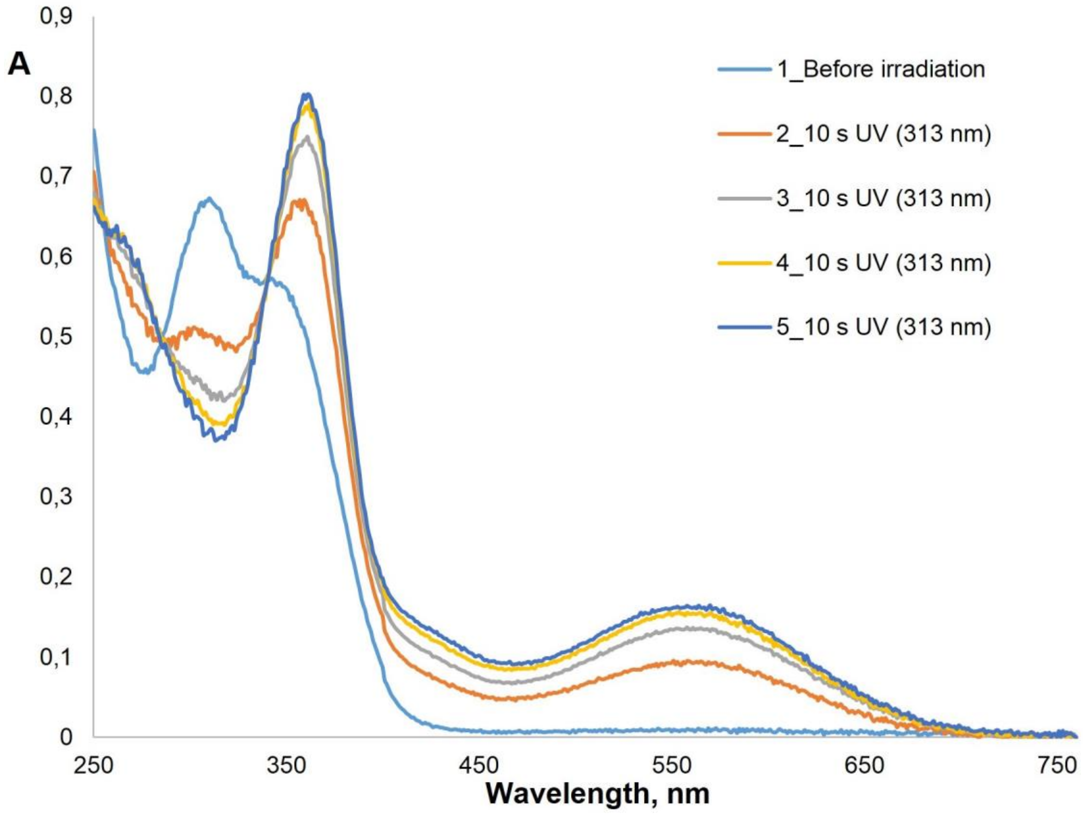

Figure S9. Electronic spectra of diarylethene **6** before and after irradiation with UV light (λ = 313 nm, acetonitrile, $c = 3.4 \times 10^{-5}$ M).

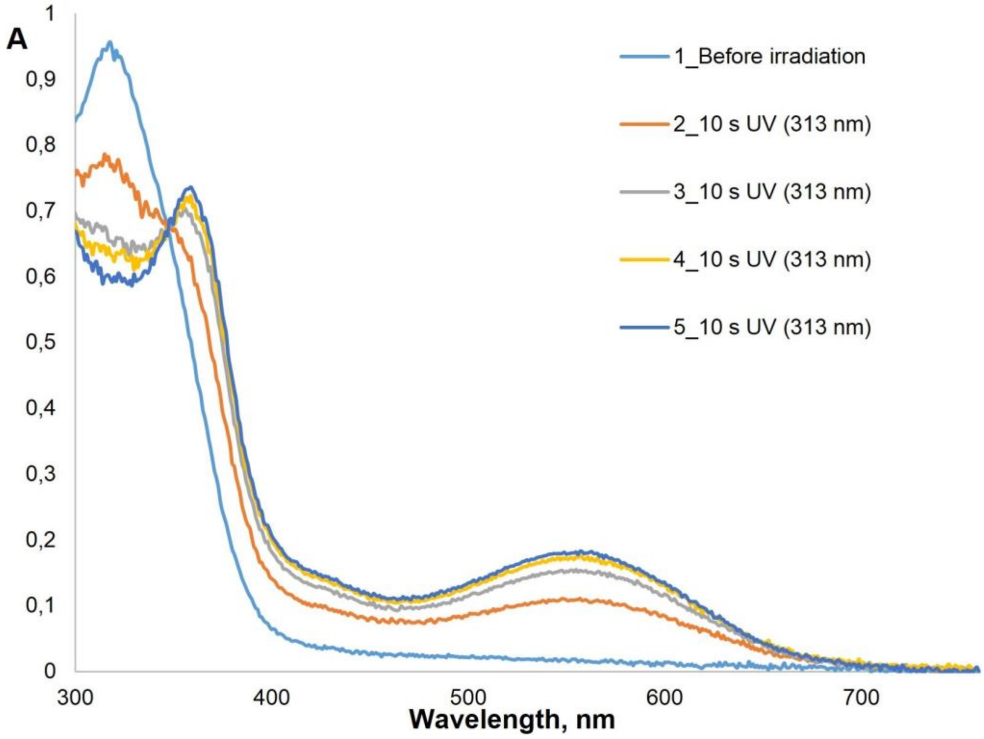

Figure S10. Electronic spectra of diarylethene **7** before and after irradiation with UV light (λ = 313 nm, toluene, $c = 3.9 \times 10^{-5}$ M).

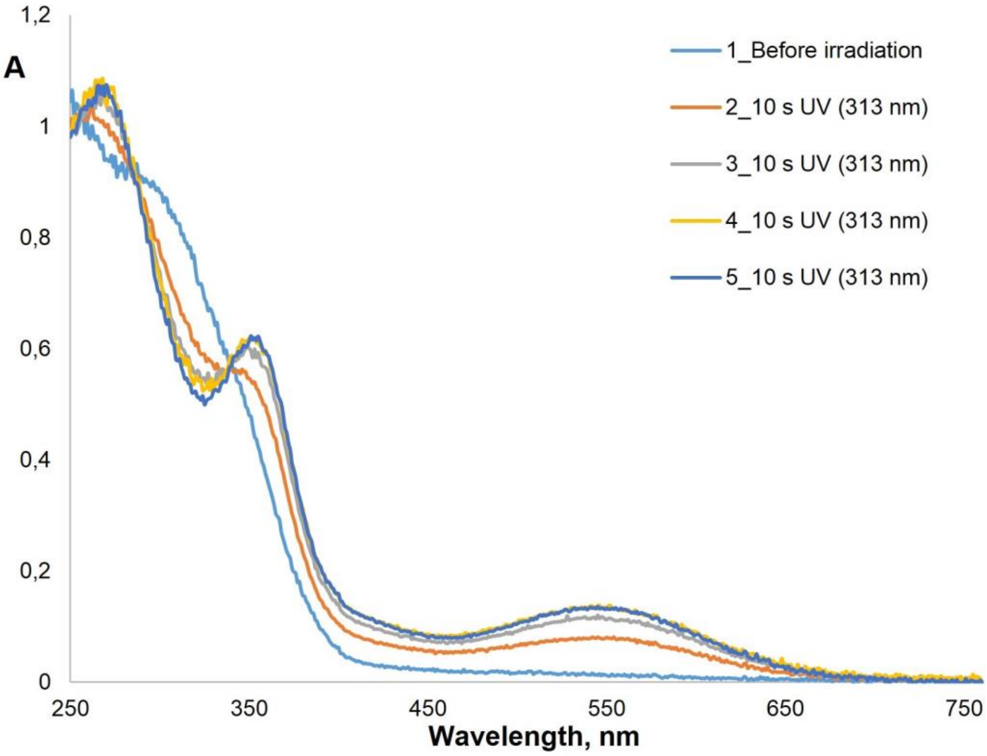

Figure S11. Electronic spectra of diarylethene **7** before and after irradiation with UV light (λ = 313 nm, acetonitrile, $c = 3.9 \times 10^{-5}$ M).

VI. ^{1}H NMR Spectra During Photochromic Performance

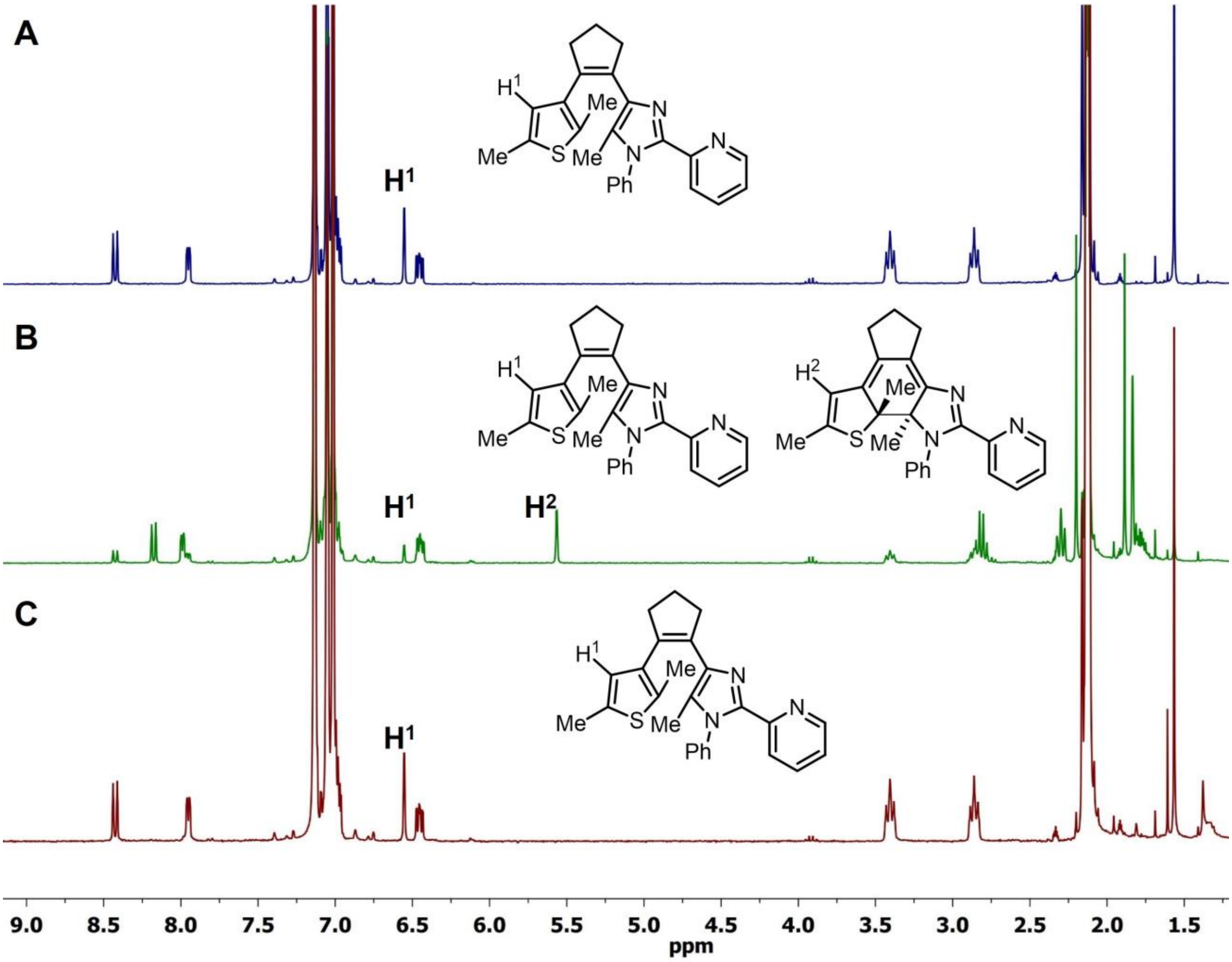

Figure S12. ^{1}H NMR spectra (toluene, c = 4.9 × 10^{-3} M) of diarylethene **4** before (A), after irradiation with UV light (λ = 365 nm, B), after irradiation with visible light (blue LED, C).

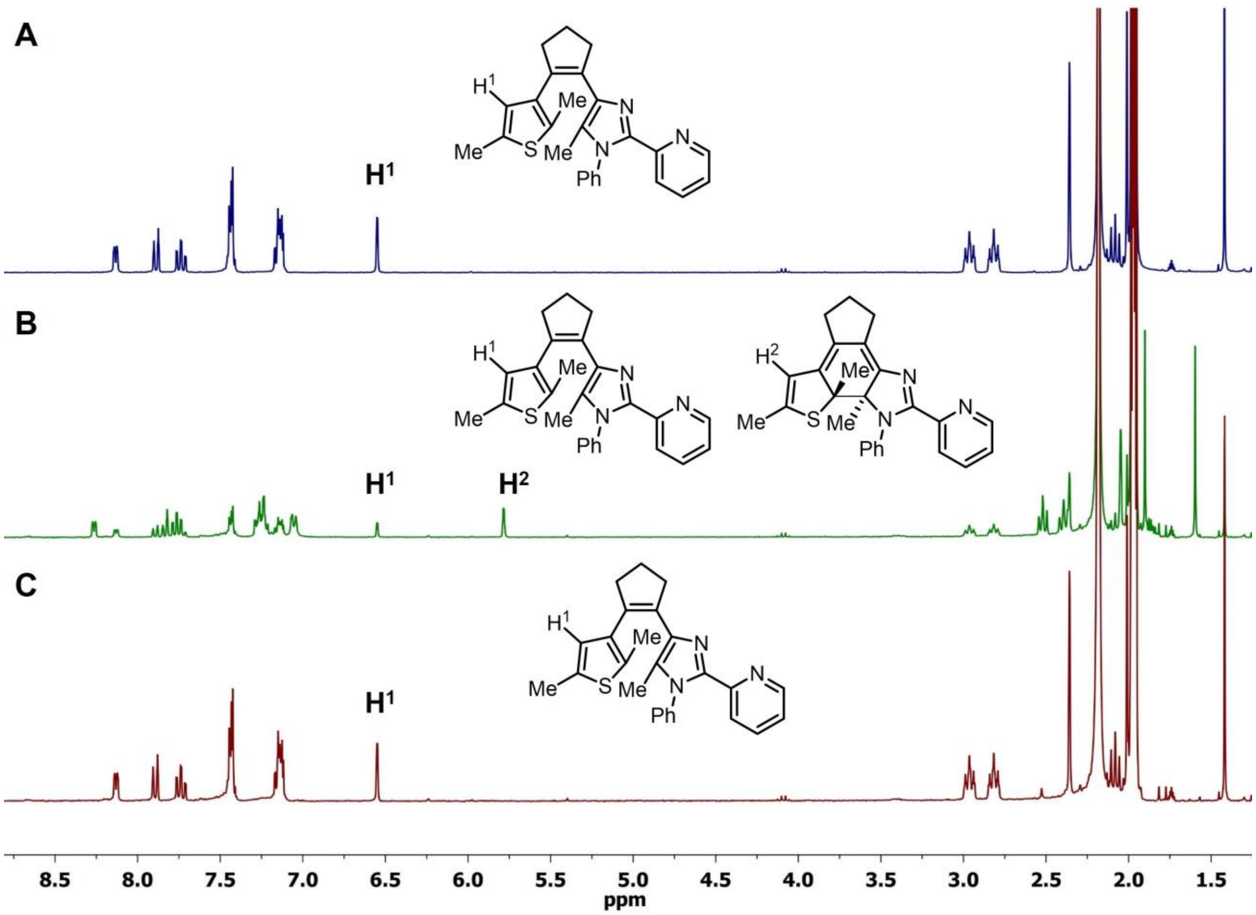

Figure S13. ^{1}H NMR spectra (acetonitrile, c = 4.9 × 10^{-3} M) of diarylethene **4** before (A), after irradiation with UV light (λ = 365 nm, B), after irradiation with visible light (blue LED, C).

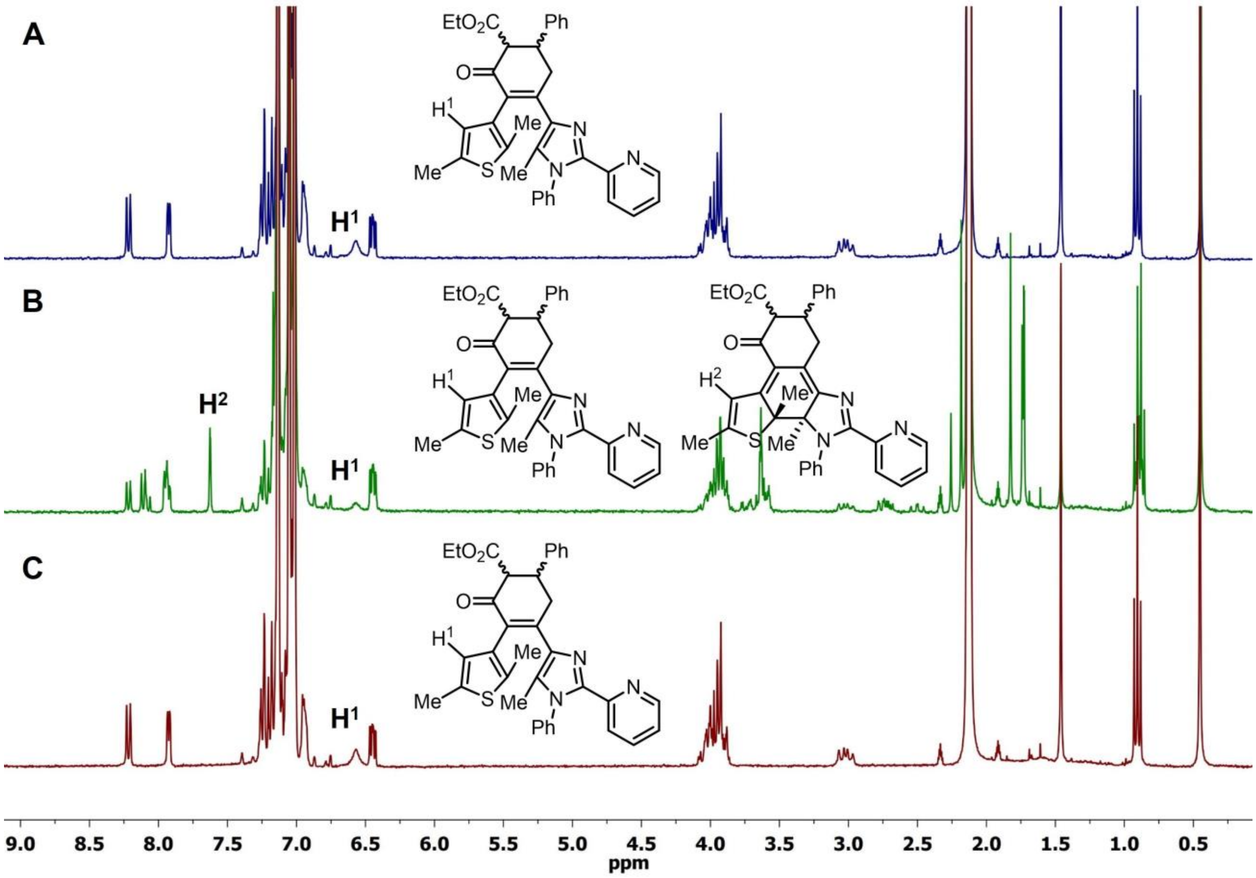

Figure S14. ^{1}H NMR spectra (toluene, c = 3.4 × 10^{-3} M) of diarylethene **6** before (A), after irradiation with UV light (λ = 365 nm, B), after irradiation with visible light (green LED, C).

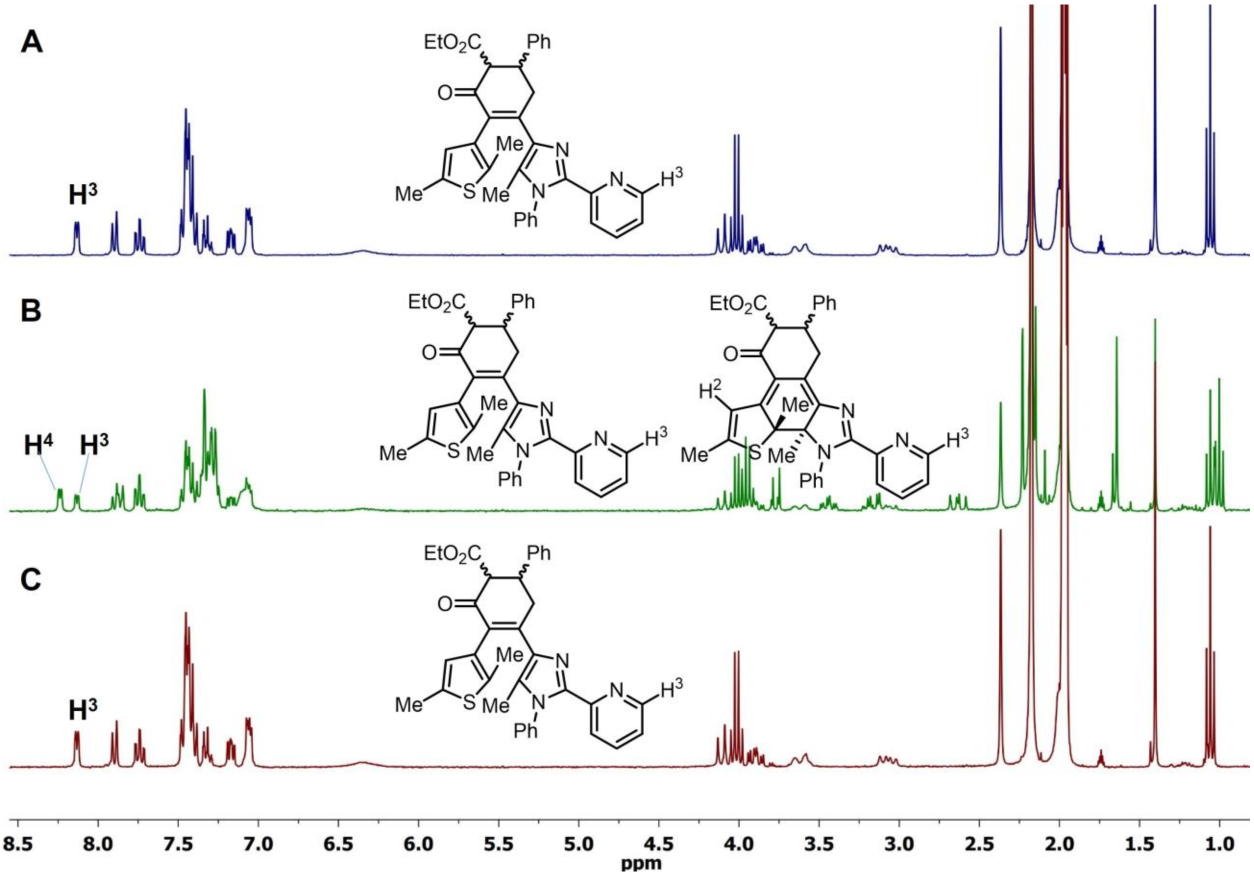

Figure S15. ^{1}H NMR spectra (acetonitrile, c = 3.4 × 10^{-3} M) of diarylethene **6** before (A), after irradiation with UV light (λ = 365 nm, B), after irradiation with visible light (green LED, C).

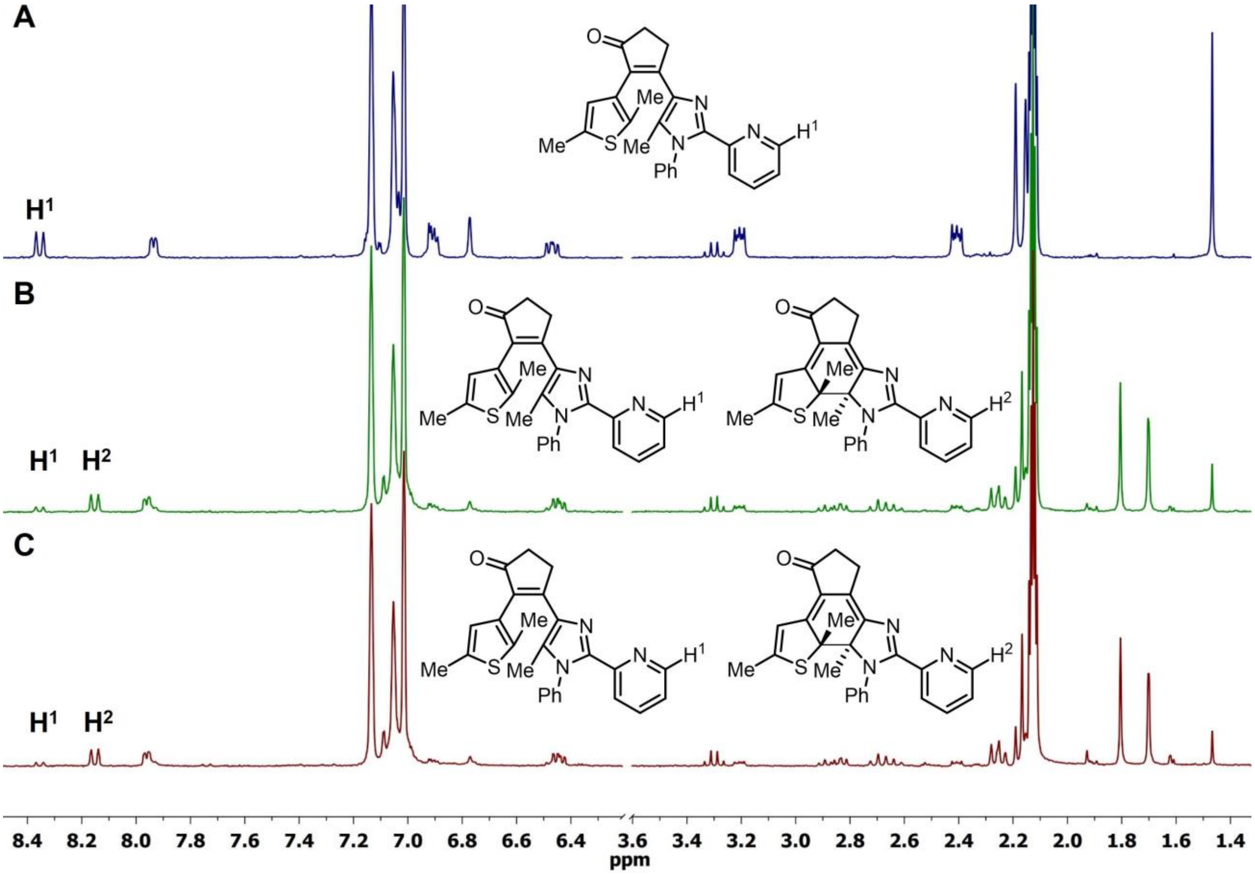

Figure S16. ^{1}H NMR spectra (toluene, $c = 3.9 \times 10^{-3}$ M) of diarylethene **3** before (A) and after (B and C) irradiation with UV light ($\lambda = 313$ nm).

VII. Studies of magnetic and spectral properties of complexes 8 and 9

Powder samples of **8** and **9** were used for variable temperature magnetic susceptibility measurements in the temperature range of 2–300 K. **8** shows a nearly constant χT product of 7.63 $cm^3mol^{-1}K$ above 125 K (Figure S17). It increases gradually upon lowering the temperature reaching a maximum of 8.80 $cm^3mol^{-1}K$ at 13 K, which is indicative of ferromagnetic coupling between the two iron(II) ions. By lowering the temperature further below 14 K, the χT product decreases sharply due to ZFS (zero-field splitting). The room temperature value of the χT product of 7.63 cm^3mol^{-1} is slightly higher than expected spin-only value for two non-interacting HS (S_{Fe} = 2) iron(II) centres ($\chi_{s.o.}T$ = 6.00 $cm^3mol^{-1}K$) due to orbital contribution. To determine the intramolecular coupling constant and g-values, magnetic data were fitted using Hamiltonian (2). The first term describes the exchange coupling, the second is the electron Zeeman effect, and the third and fourth are axial ZFS terms for each iron(II) ion.

$$\hat{\mathbf{H}} = -2J\hat{\mathbf{S}}_1\hat{\mathbf{S}}_2 + \mu_B(g_1\hat{\mathbf{S}}_1 + g_2\hat{\mathbf{S}}_2)\boldsymbol{B} + D_1\left(\hat{\mathbf{S}}_{1z}^2 - \frac{1}{3}S_1(S_1+1)\right) + D_2\left(\hat{\mathbf{S}}_{2z}^2 - \frac{1}{3}S_2(S_2+1)\right) \quad (2)$$

Due to the symmetry of the complex, $D_1 = D_2$ and $g_1 = g_2$ are used in the simulation. The fit affords a ferromagnetic coupling constant J = +0.5 cm^{-1} and D = 5.0 cm^{-1}. The determined $g_1 = g_2$ = 2.24 are in the expected range for d^6 systems. To get further insight, field dependent magnetic measurements from 0 to 7 T at 2 K were conducted (see Figure S18). At fields > 4 T eight Bohr magnetons per mole ($N_A\mu_B$) correspond to two ferromagnetically coupled HS iron(II) centres.

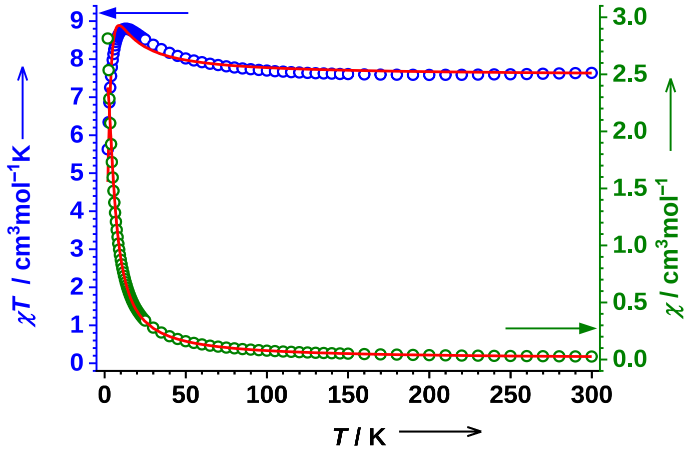

Figure S17: Variable temperature χT product (blue) and χ (green) of **8** measured on a powder sample at an external magnetic field of 1 T in the heating mode (1 K min^{-1}, 0.5 K intervals between 2–25 K, 5 K intervals between 25–150 K and 10 K intervals between 150–300 K). See the text for fitting parameters.

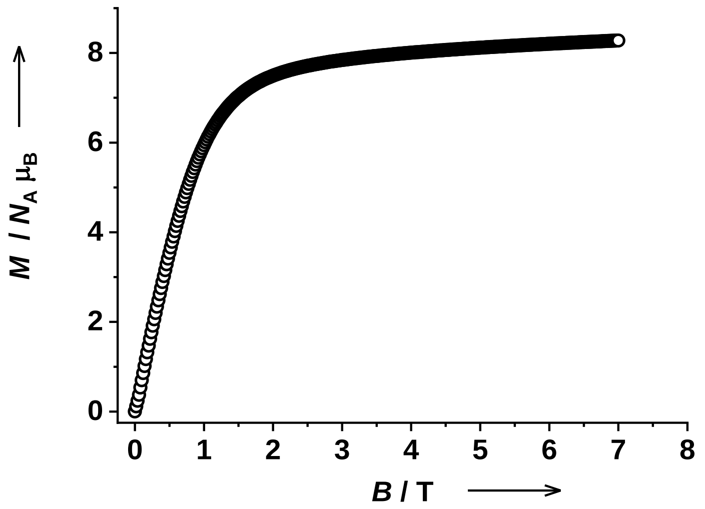

Figure S18: Field-dependent reduced magnetization of **8** measured at 2.0 K.

The χT product of **9** increases to almost constant value of 14.61 $cm^3mol^{-1}K$ at room temperature, which is in good agreement with four uncoupled high-spin iron(II) ions ($\chi_{s.o.}T$ = 12.00 $cm^3mol^{-1}K$) (Figure S19). A large drop in susceptibility is observed at temperatures below 40 K due to ZFS. The magnetic data were fitted using Hamiltonian (3), with the first term describing the electron Zeeman effect, whereas the second term is due to the ZFS.

$$\hat{\mathbf{H}} = \sum_{i=1}^{4} \left[\mu_B g_i \hat{\mathbf{S}}_i \boldsymbol{B} + D_i \left(\hat{\mathbf{S}}_{iz}^2 - \frac{1}{3} S_i (S_i + 1) \right) \right] \qquad (3)$$

The simplifications $g_1 = g_2 = g_3 = g_4$ and $D_1 = D_2 = D_3 = D_4$ have been assumed to exclude overparamaterization, which yields the fitting parameters for g = 2.20 and D = 5.9 cm^{-1}, which is in agreement with a d^6-system. Field-dependent magnetic measurements show a more gradual increase of magnetization compared to **8**. The magnetization does not saturate at 7 T (Figure S20). The observed maximum of 14.17 $N_A\mu_B$ at 7 T points to four S_{Fe} = 2 ions (16 $N_A\mu_B$ is expected).

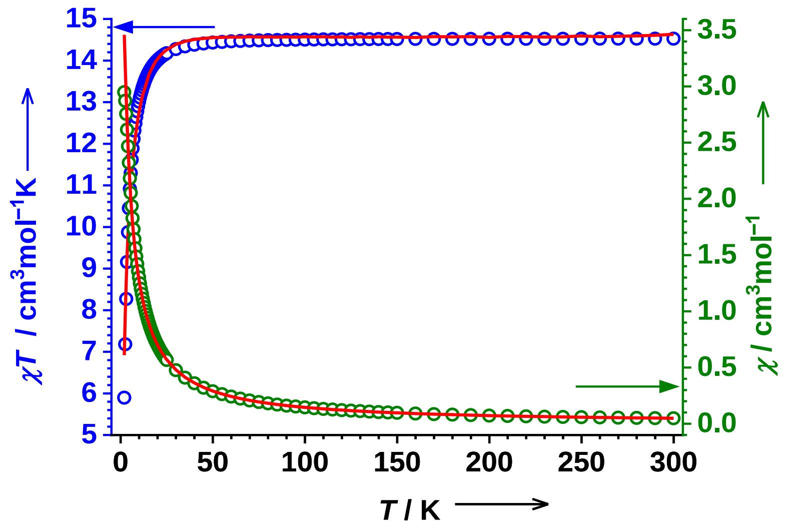

Figure S19: Variable temperature χT product (blue) and χ (green) of **9** measured on a powder sample at an external magnetic field of 1 T in the heating mode (1 K min^{-1}, 0.5 K intervals between 2–25 K, 5 K intervals between 25–150 K and 10 K intervals between 150–300 K). See the text for fitting parameters.

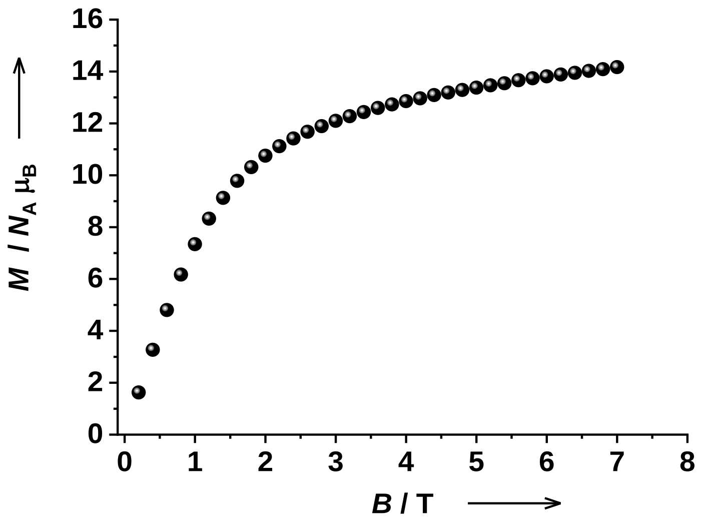

Figure S20: Field-dependent reduced magnetization of **9** measured at 2.0 K.

Mössbauer spectroscopy

Zero-field ^{57}Fe Mössbauer spectra were obtained on a powder sample of **8** at 77 K and room temperature (rt). The rt spectrum reveals a quadrupole doublet with an isomer shift (δ) of 0.89 mm s^{-1} and a quadrupole splitting ($|\Delta E_Q|$) of 2.64 mm s^{-1} which is assigned to a HS iron(II) ion (Figure S21, top) [S12]. Cooling to 77 K induces no change in spin state, as the spectrum (Fig S21, bottom) is still appearing phenotypically similar to the room temperature data. The minor changes in the isomer shift (δ) of 1.01 mm s^{-1} and a quadrupole splitting ($|\Delta E_Q|$) of 2.86 mm s^{-1} are in good agreement with HS iron(II).

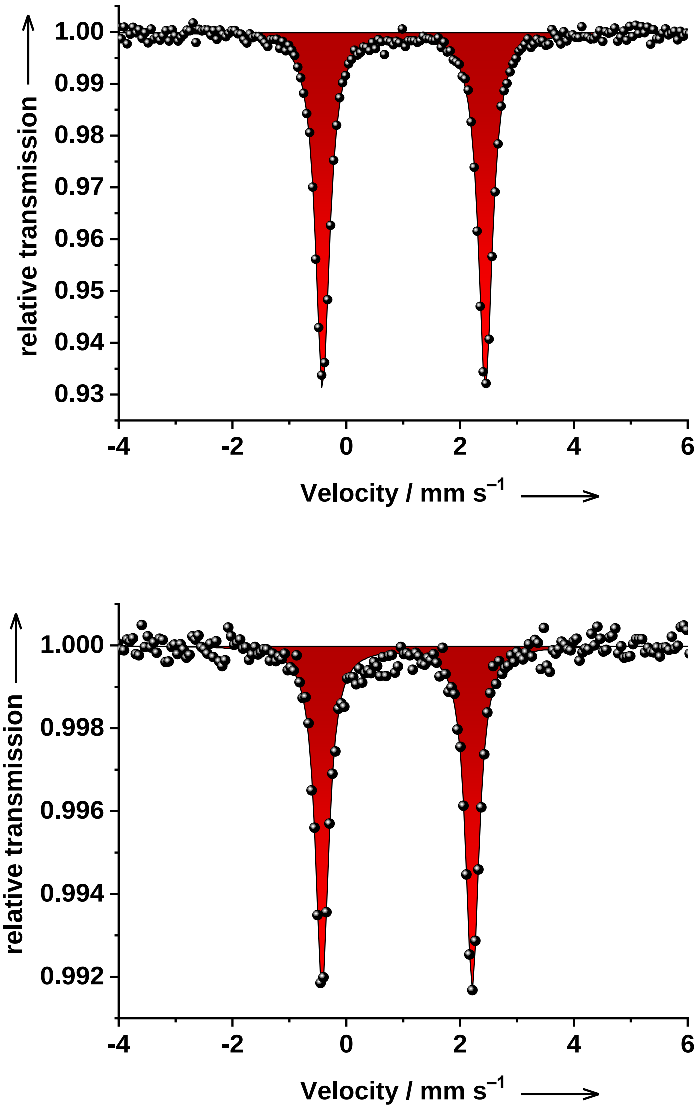

Figure S21: Zero-field ^{57}Fe Mössbauer spectra measured on **8** at 77 K (top) and 297 K (bottom).

Electronic absorption spectroscopy

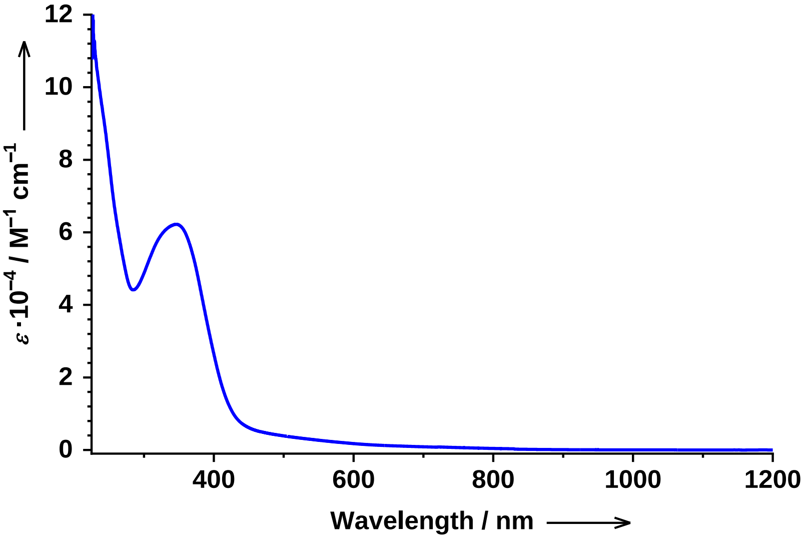

Figure S22: Electronic absorption spectra of **8** recorded in a DCM solution (c = 3 · 10^{-5} M) at rt.

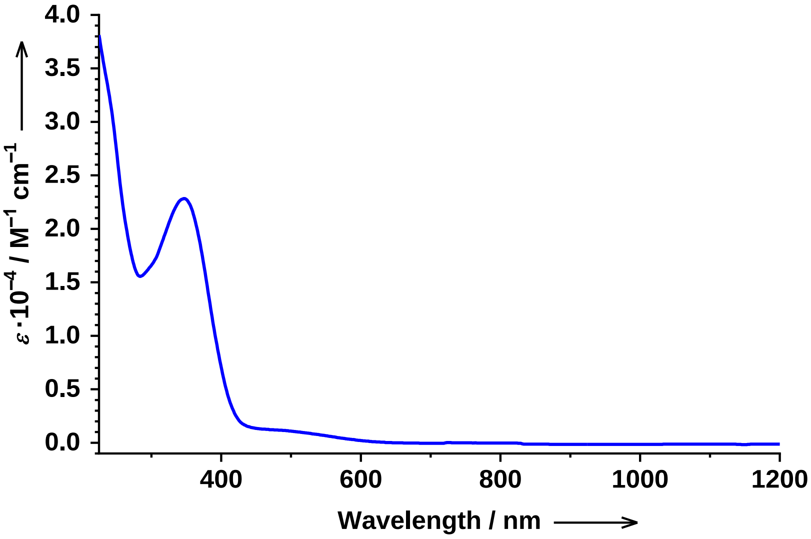

Figure S23: Electronic absorption spectrum of **9** recorded in a DCM solution (c = 3 · 10^{-5} M) at rt.

VIII. Copies of ^{1}H and ^{13}C NMR spectra

^{1}H NMR spectrum for compound 1

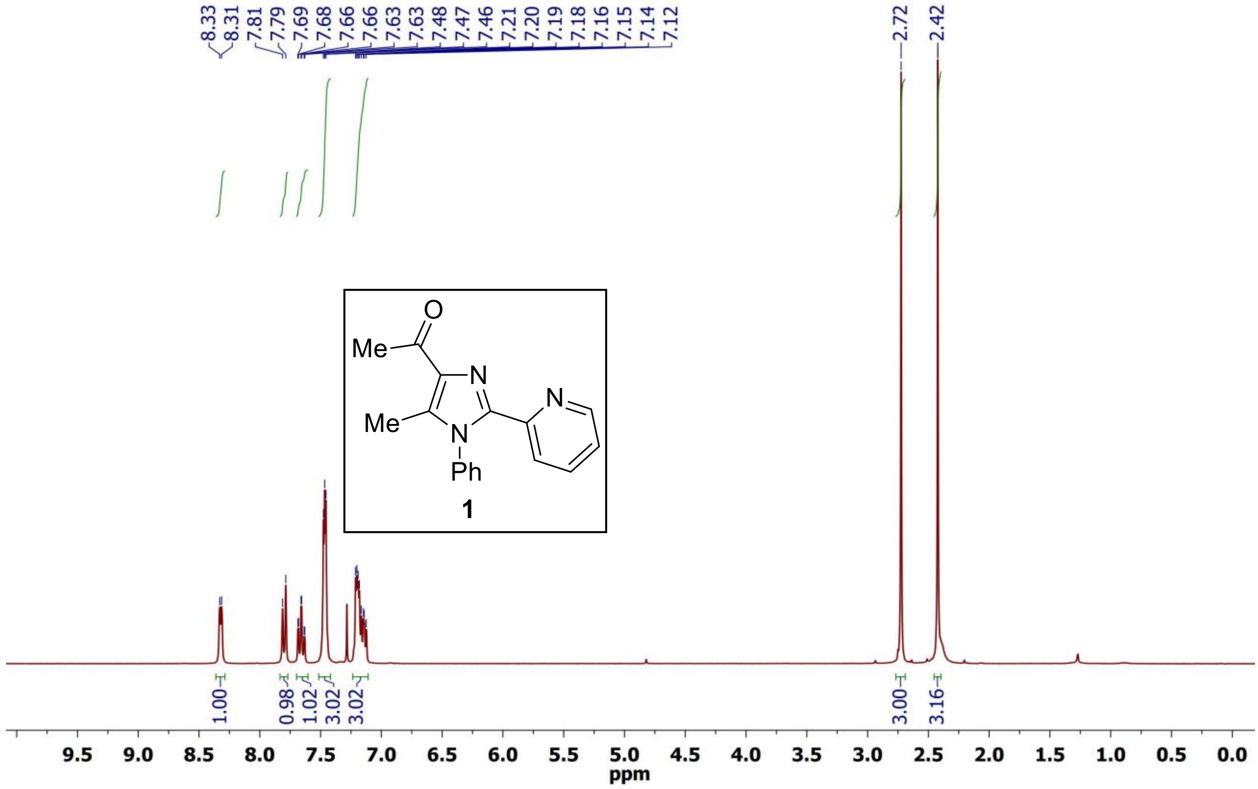

^{13}C NMR spectrum for compound 1

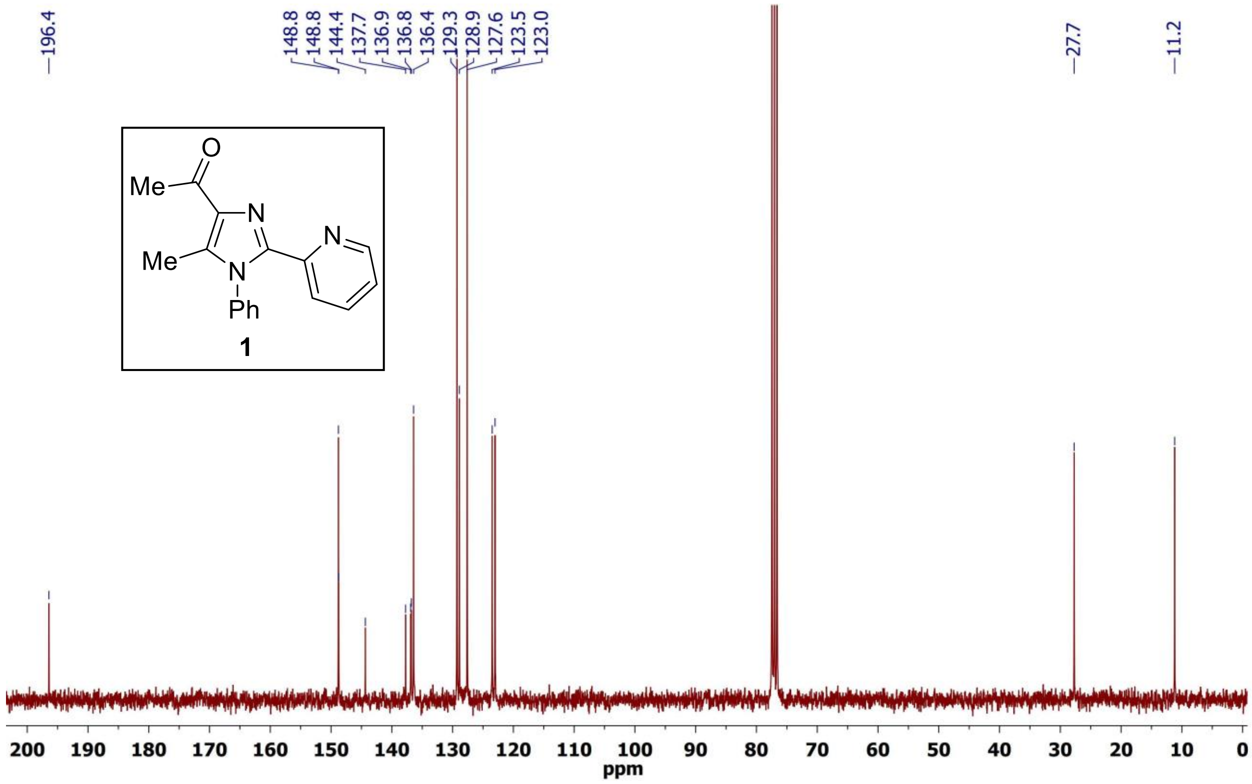

^{1}H NMR spectrum for compound 3

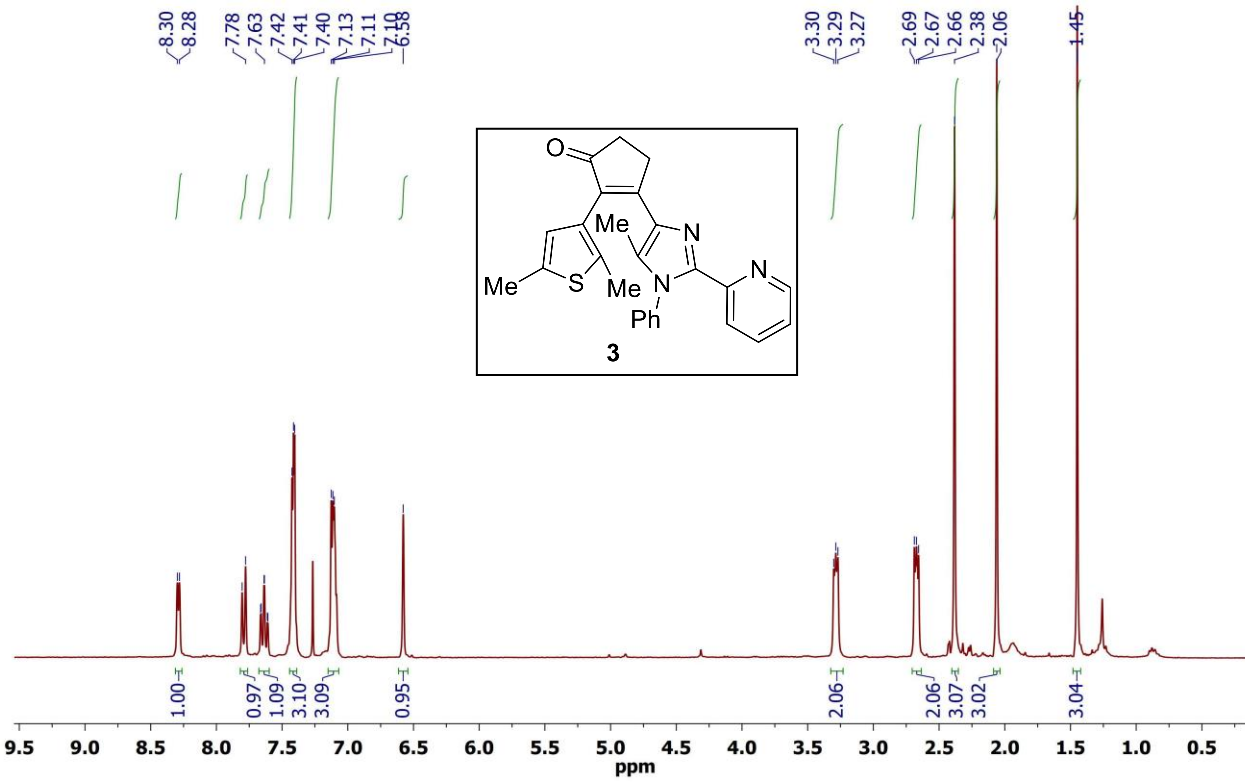

^{13}C NMR spectrum for compound 3

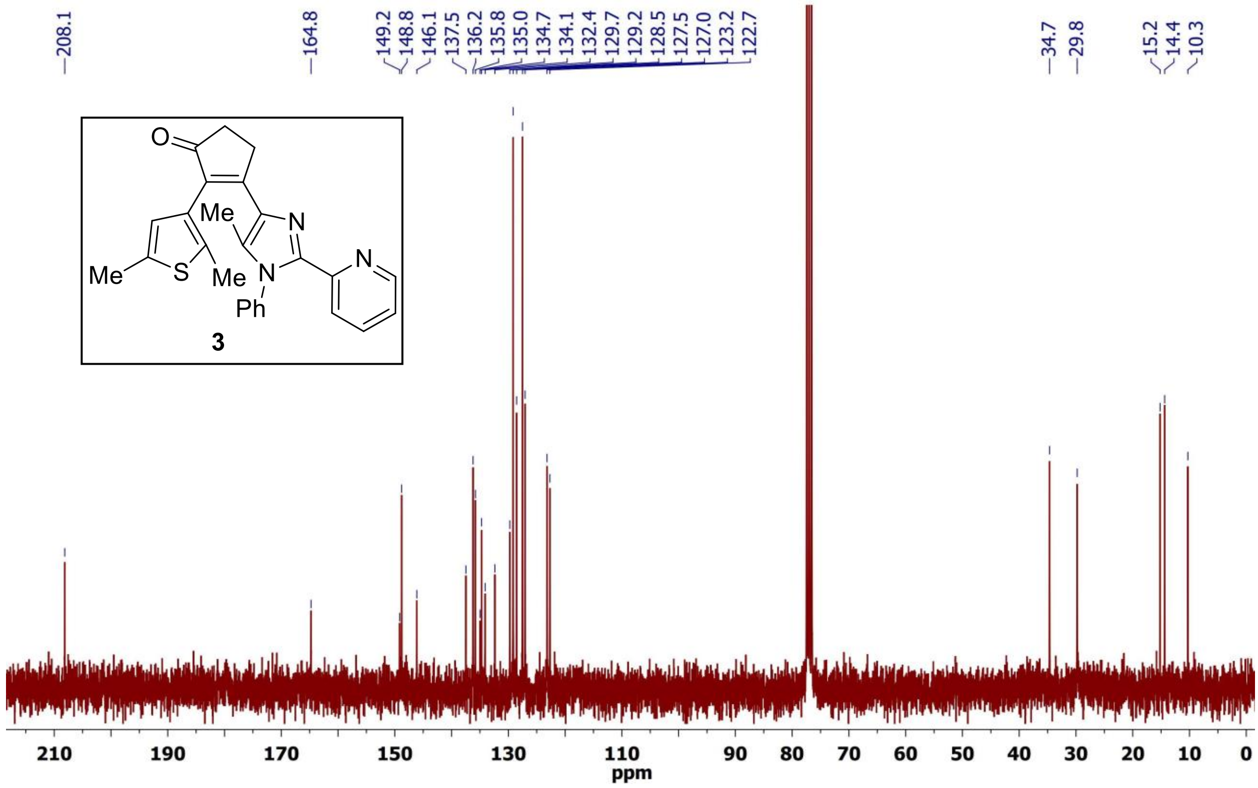

^{1}H NMR spectrum for compound 4

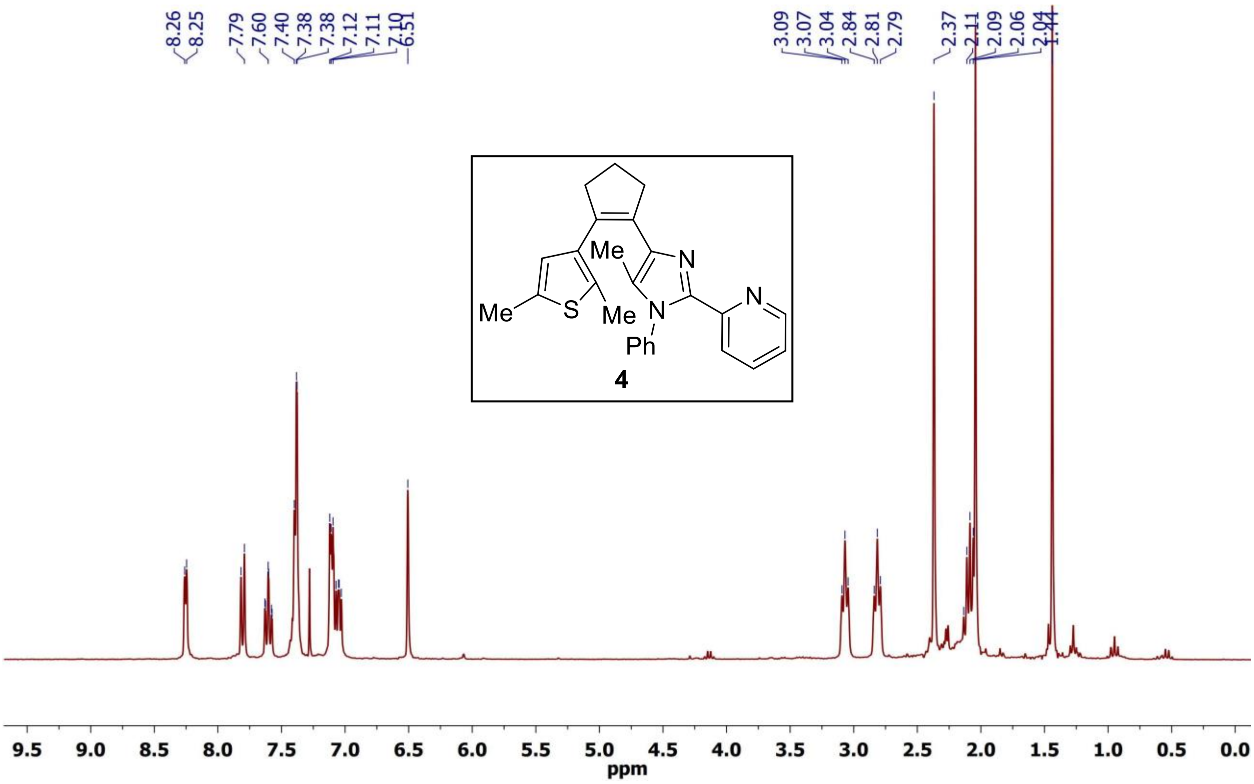

^{13}C NMR spectrum for compound 4

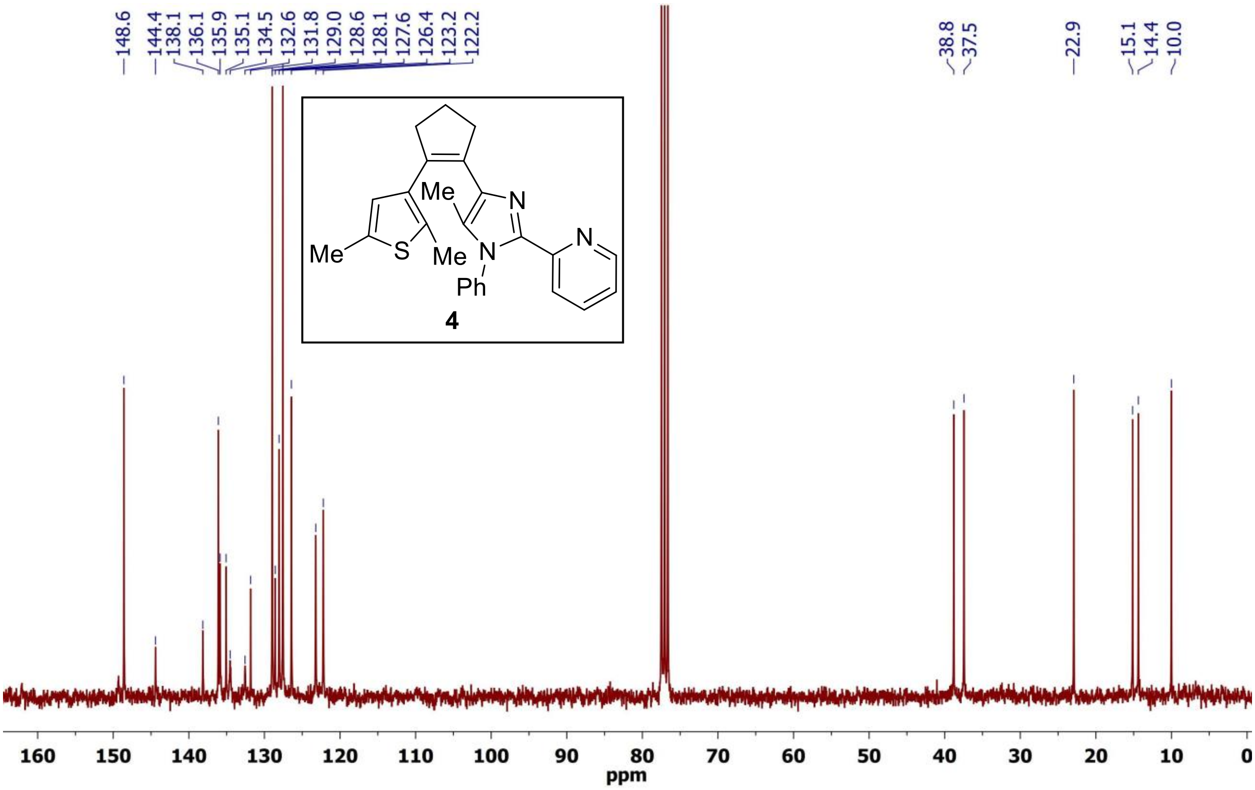

^{1}H NMR spectrum for compound 5

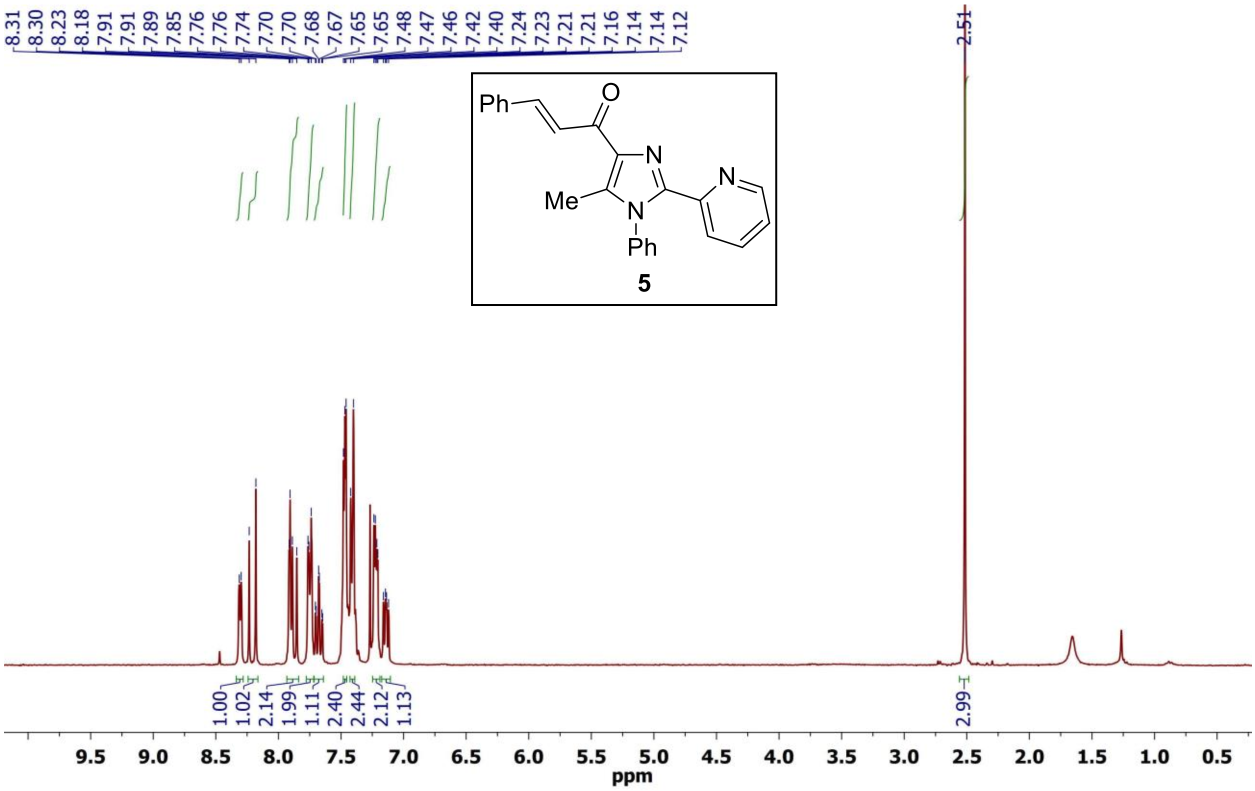

^{13}C NMR spectrum for compound 5

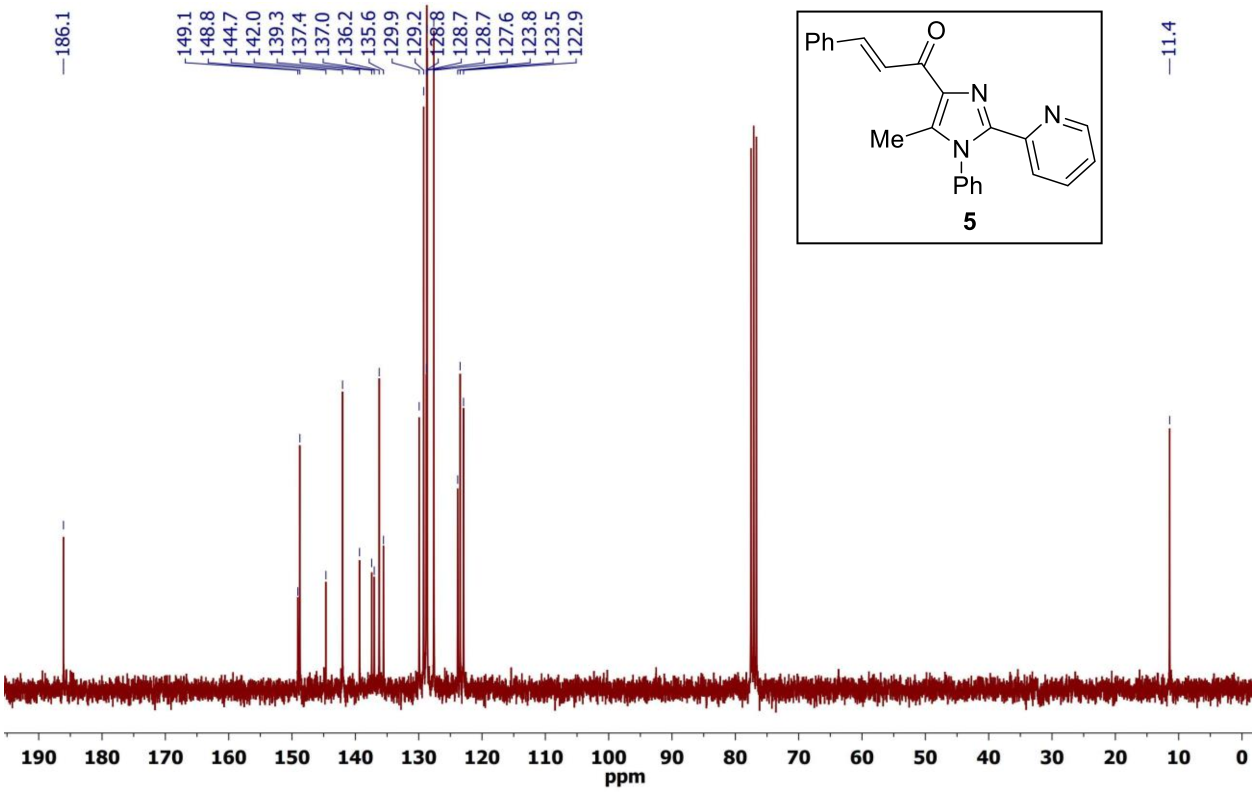

^{1}H NMR spectrum for compound 6

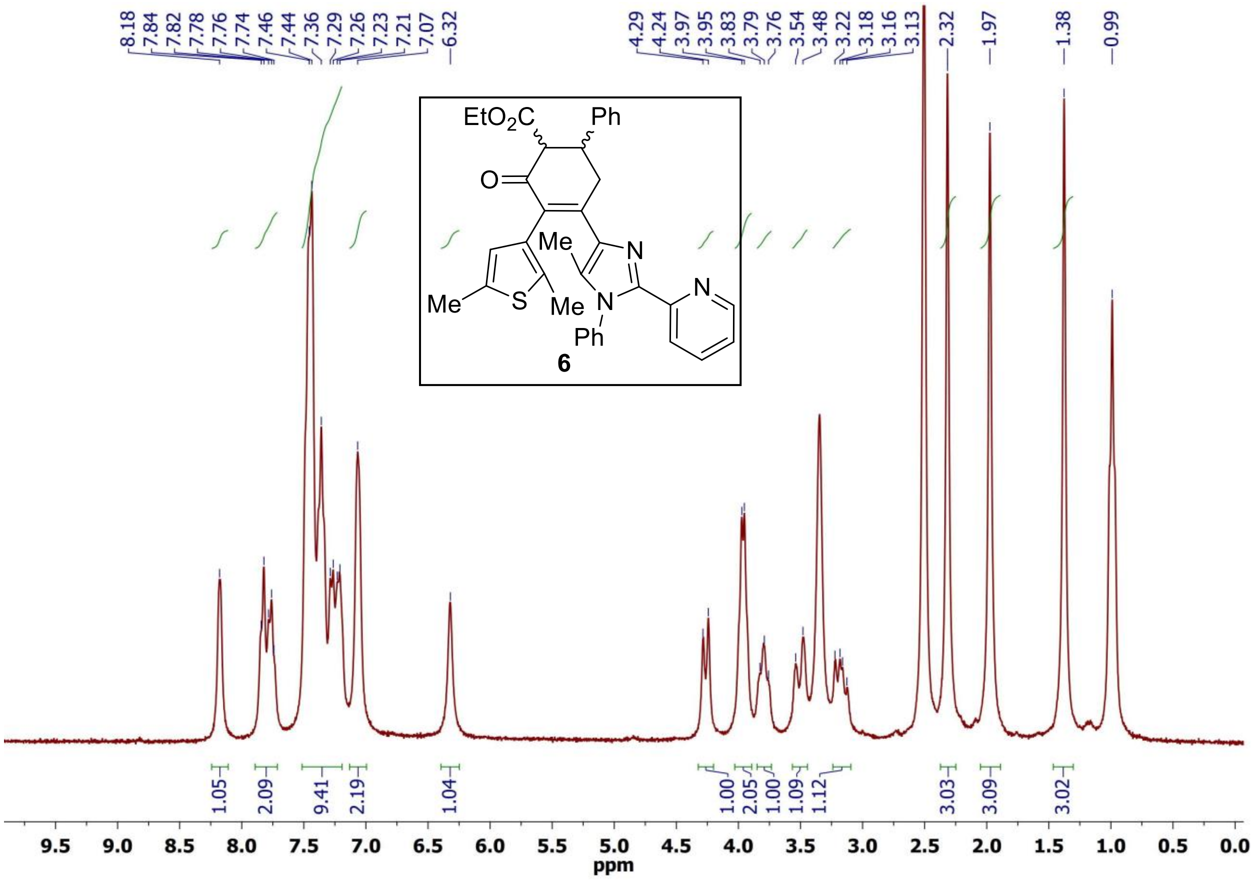

^{13}C NMR spectrum for compound 6

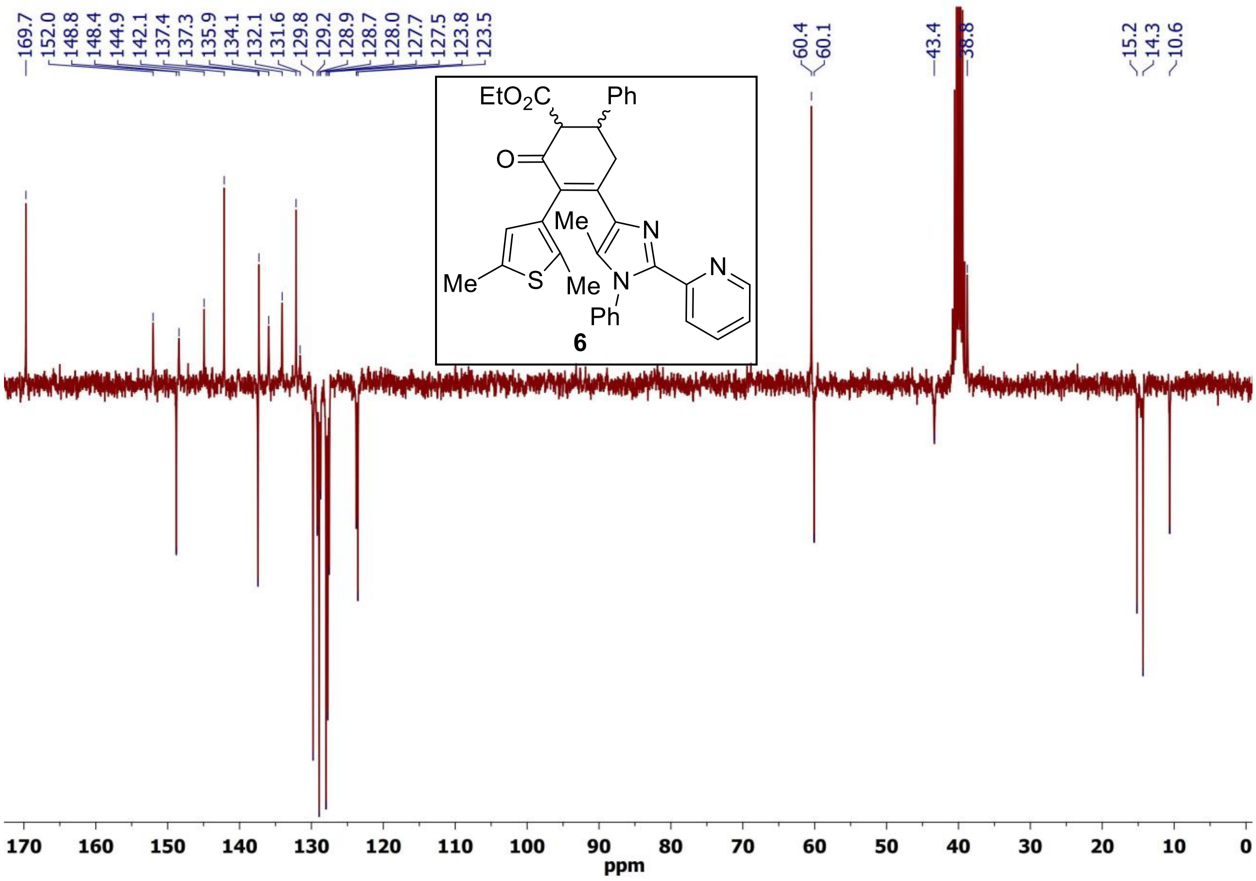

^{1}H NMR spectrum for compound 7

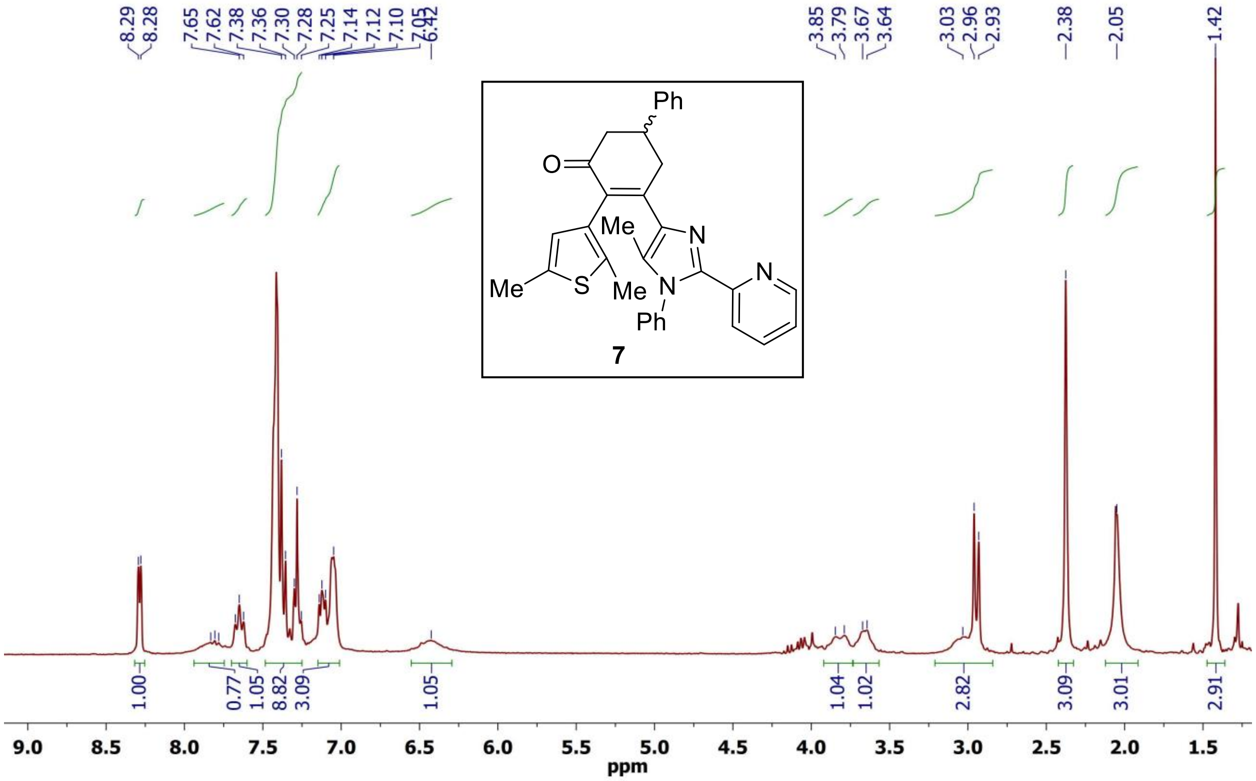

^{13}C NMR spectrum for compound 7

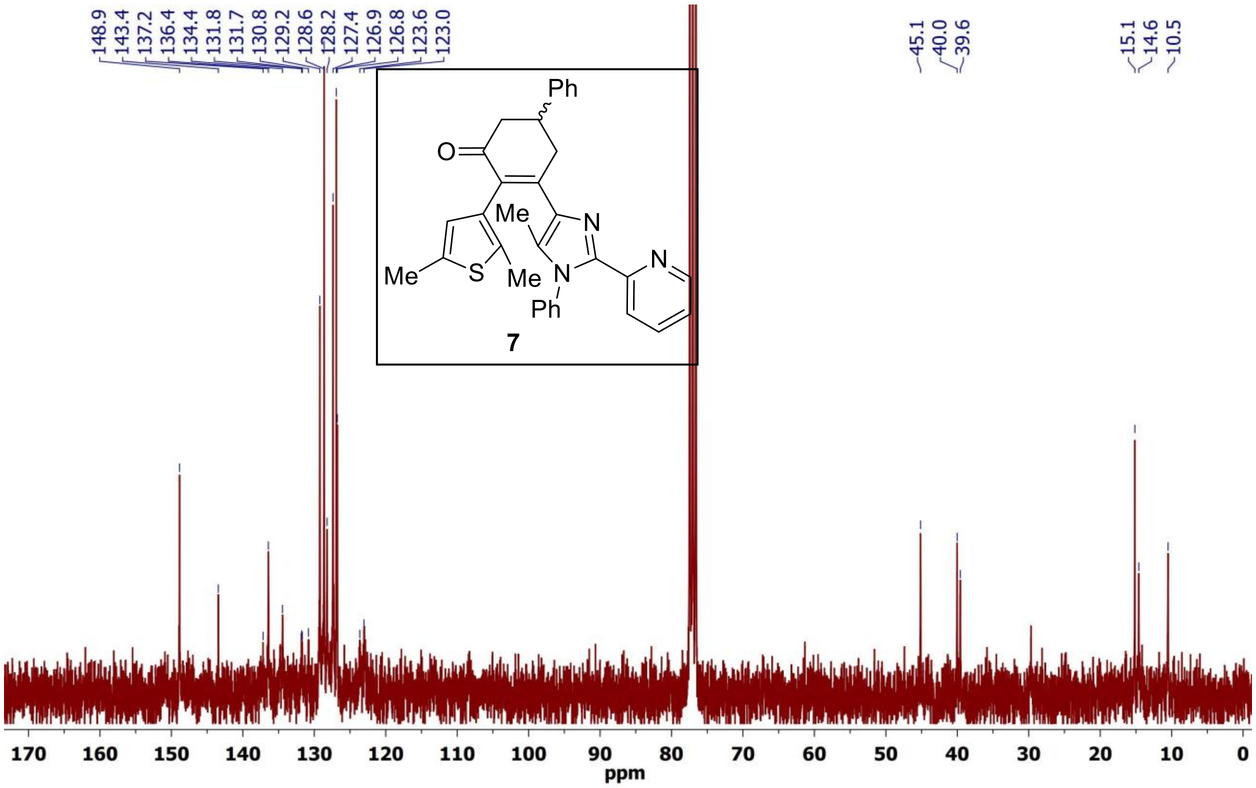

IX. References

[S1] Sumi, T.; Takagi, Y.; Yagi, A.; Morimoto, M.; Irie, M. *Chem. Commun.* **2014**, *50*, 3928-3930.

[S2] SADABS 2008/1, Bruker AXS, Inc.: Madison, WI, 2009.

[S3] Sheldrick, G. *Acta Cryst. A* **2008,** *64*, 112-122.

[S4] Sheldrick, G. Acta Cryst. C **2015**, *71*, 3-8.

[S5] Kahn, O., *Molecular Magnetism*. VCH Publishers Inc., 1993.

[S6] Chilton, N. F.; Anderson, R. P.; Turner, L. D.; Soncini, A.; Murray, K. S. *J. Comput. Chem.* **2013,** *34*, 1164-1175.

[S7] E. Bill, MFIT, version 1.1, MPI for Bioinorganic Chemistry, Mülheim/Ruhr, Germany, 2008

[S8] Shimkin, A. A.; Shirinian, V. Z.; Mailian, A. K.; Lonshakov, D. V.; Gorokhov, V. V.; Krayushkin, M. M. *Russ. Chem. Bull.* **2011**, *60*, 139−142.

[S9] Lvov, A. G.; Shirinian, V. Z.; Zakharov, A. V.; Krayushkin, M. M.; Kachala, V. V.; Zavarzin, I. V. *J. Org. Chem.* **2015**, *80*, 11491-11500.

[S10] Lvov, A. G.; Kavun, A. M.; Kachala, V. V.; Nelyubina, Y. V.; Metelitsa, A. V.; Shirinian, V. Z. *J. Org. Chem.* **2017**, *82*, 1477–1486.

[S11] Milek, M.; Heinemann, F. W.; Khusniyarov, M. M. *Inorg. Chem.* **2013**, *52*, 11585-11592.

[S12] Gütlich, P.; Bill, E.; Trautwein, A. X., *Mössbauer Spectroscopy and Transition Metal Chemistry*. Springer-Verlag: Berlin - Heidelberg, 2011.
